# Distribution of Legacy and Emerging PFASs in a Terrestrial Ecosystem Located near a Fluorochemical Manufacturing Facility

**DOI:** 10.3390/toxics13080689

**Published:** 2025-08-19

**Authors:** Jodie Buytaert, Marcel Eens, Lieven Bervoets, Thimo Groffen

**Affiliations:** 1 ECOSPHERE, Department of Biology, University of Antwerp, Groenenborgerlaan 171, 2020 Antwerp, Belgium; lieven.bervoets@uantwerpen.be (L.B.); thimo.groffen@uantwerpen.be (T.G.); 2 Behavioural Ecology and Ecophysiology Group, Department of Biology, University of Antwerp, Universiteitsplein 1, 2610 Wilrijk, Belgium; marcel.eens@uantwerpen.be

**Keywords:** biomonitoring, songbirds, terrestrial invertebrates, per- and polyfluoroalkyl substances

## Abstract

This study investigated the distribution of 29 legacy and emerging per- and polyfluoroalkyl substances (PFASs) in soil, nettles, invertebrates, and plasma and feathers of great tits (*Parus major*) of a terrestrial ecosystem near a fluorochemical plant. Additionally, the vertical distribution of PFASs in soil was assessed, as well as taxon-specific differences among terrestrial invertebrate species. Finally, associations between soil and biota, and among biological matrices, were assessed. Most accumulation profiles were dominated by long-chained PFASs, mainly perfluorooctane sulfonic acid (PFOS), while short-chained PFASs were less detected. Long-chained perfluoroalkyl carboxylic acids (PFCAs) adsorbed in the upper soil layers, while short-chained PFAS and perfluoroalkyl sulfonic acids (PFSAs) tended to migrate deeper. The several taxon-specific differences were likely due to dietary differences. Significant associations, especially for long-chained PFCAs and PFOS, were found among most matrices. This indicates that (1) these PFASs found in these matrices are most likely originating from the same pollution source, (2) there is a possible transfer of these PFASs between matrices, (3) there is bioaccumulation from one to another matrix, and (4) some matrices might be used as proxies to estimate PFAS concentrations in other terrestrial matrices. Finally, feathers accumulated more PFASs than plasma, as they were most likely exposed through different routes of exposure and PFAS affinity. Therefore, they are not suitable for internal PFAS monitoring but can provide complementary information about the exposure and about the presence/absence of PFASs in certain habitats.

## 1. Introduction

Over the last decades, per- and polyfluoroalkyl substances (PFASs) have emerged as substances used in many different industrial and commercial applications [1,2,3]. This has resulted in increasing concentrations of these chemicals in the environment. Due to their unique physicochemical properties, most PFASs are very persistent in the environment and bioaccumulative [4]. Furthermore, soils are known to be sinks for many organic pollutants, including PFASs [5]. PFASs can be adsorbed to the soil through different mechanisms, with electrostatic and hydrophobic interactions being the most important [6]. In addition, PFASs become more hydrophobic with increasing carbon chain length, which results in higher sorption rates for longer-chain PFASs [7]. However, short-chain PFASs can also be present in high concentrations, but they most likely migrate deeper within the pore water and groundwater due to their higher hydrophilicity [8]. The transformation of several precursor compounds into shorter and more mobile PFASs can also result in the presence of shorter-chained PFASs within the soil [9].

PFAS concentrations in the wild tend to be higher surrounding hotspots, and PFAS composition profiles tend to differ distinctly depending on the source of contamination, the matrices that were investigated, and the year of sampling [10,11,12,13,14,15]. Therefore, it is important to monitor the behaviour and distribution of these compounds in such environments, to be able to regulate and/or remediate these PFASs in the environment. Most studies have investigated the distribution within the aquatic environment, but terrestrial field studies including different species and emerging PFASs remain scarce [16,17,18,19,20]. Therefore, there is an urgent need for studies on emerging PFASs in terrestrial ecosystems near hotspots to better understand the behaviour of these emerging PFASs in such environments, given the uncertainty regarding the specific PFASs present across different environmental matrices and their respective concentrations, as reviewed by Gkika et al. [21].

Additionally, studies on the vertical distribution of emerging PFASs in soil remain scarce [14,22,23,24], which makes it still unclear whether emerging PFASs will reside in topsoil or eventually migrate deeper. Therefore, there is an urgent need for more research regarding the behaviour of emerging PFASs in soils. Additionally, studies on the accumulation of PFASs in plants have mostly focused on crops and legacy PFASs due to the risk for human exposure [24,25,26]. Other terrestrial plants have only been limitedly studied and, therefore, it is unclear whether other organisms, besides humans, are also being exposed to PFASs present in terrestrial plants [7,16]. In addition, differences in PFAS accumulation among terrestrial invertebrates have, to the best of our knowledge, only rarely been studied. Most of the few studies on invertebrates were conducted under laboratory conditions, as reviewed by Ankley et al. [27] and Gkika et al. [21]. Additionally, field studies regarding the accumulation of PFASs within terrestrial invertebrates remain scarce [16,19,28,29,30,31,32,33]. Furthermore, PFASs tend to biomagnify through the food chain, making it important to include higher trophic level organisms in biomonitoring studies. Moreover, the use of songbirds in terrestrial studies can provide additional information regarding local PFAS contamination. An example of a songbird species that is a good bioindicator for monitoring site-specific contamination is the great tit (*Parus major*) [3,34,35]. Their blood plasma is useful for monitoring local contamination due to their relatively small home ranges [36], presence in urban and industrial areas, and preference to sleep and breed in nestboxes. For now, many studies investigating PFAS accumulation in birds rely on destructive and/or invasive sampling methods. Thus, there is still a need for more studies to investigate the suitability of bird feathers as a non-destructive biomonitoring tool, as mentioned by Jaspers et al. [37], since feathers are more likely to reflect exposure up until the last moult. Additionally, it is unknown whether other terrestrial matrices could be used as a proxy to monitor the PFAS contamination in higher vertebrates.

Therefore, this study aimed to investigate the differences and similarities in PFAS accumulation and profiles among several matrices of a simplified terrestrial ecosystem (soil, stinging nettles (*Urtica dioica*), multiple invertebrate taxa (isopods, earthworms, snails, slugs, and spiders), and birds (great tit, *Parus major*)). More specifically, this study aimed to investigate how PFAS profiles vary among these matrices, whether PFAS levels in soil and biota were associated across the different matrices of the terrestrial ecosystem, and whether feathers can serve as a non-destructive proxy for internal PFAS exposure in birds. We hypothesised that PFAS concentrations would vary substantially among terrestrial matrices due to different routes of exposure, different diets between animals, and the different positions in the food chain. Furthermore, we hypothesised that PFAS concentrations in soil and biota would be associated mostly with the soil invertebrates, whilst nettles would be associated mostly with the herbivorous invertebrates. Another hypothesis was that PFAS concentrations in spiders would have the most associations with those in great tit plasma, due to their predator–prey relationship. Finally, we expected that PFAS concentrations in feathers only represent those in plasma to a limited extent, due to them representing accumulation over different time spans.

## 2. Materials and Methods

### 2.1. Study Area and Sample Collection

**Figure 1:**
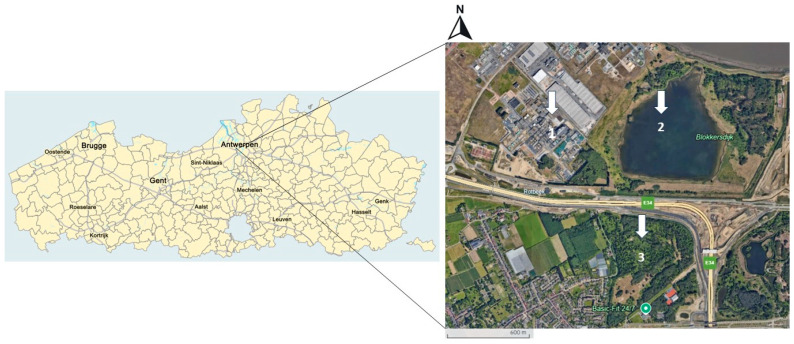
Overview of the two sampling locations, Blokkersdijk/3M and Vlietbos, located in Flanders, Belgium. Inset shows more specifically the Antwerp region (1 = 3M site, 2 = Blokkersdijk, 3 = Vlietbos, sampling sites 1 and 2 were considered as one sampling location). Point (1) is a plant site with some adjacent trees surrounding the property, (2) is a nature reserve adjacent to the plant site where birds have the occasion to breed, forage, and sleep, and (3) is a public forest area located on the other side of a large highway than the other two sites.

The study was conducted at two different sampling sites near a fluorochemical manufacturing facility, 3M in Zwijndrecht, Belgium (Figure 1).

The two sites consisted of (1) the combined site of 3M Zwijndrecht itself with the neighbouring site of Blokkersdijk (a nature reserve, property of Natuurpunt (an independent volunteer association that ensures the protection of vulnerable and endangered nature in Flanders)) and (2) Vlietbos (a public forest area, maintained by the Agency for Nature and Forest and Natuurpunt). Before conducting the sampling, the sites of 3M and Blokkersdijk were considered as individual sites with 20 and 18 nest boxes, respectively, but after we observed that the same individually marked great tits foraged in both sites and slept in nest boxes at both sites, these sites were considered as one site. The site of 3M is an industrial plant site, with some green areas surrounding the plant. Blokkersdijk is a nature reserve and is a foraging and breeding habitat for many birds and is only partially accessible to the public. At last, Vlietbos is a public forest area at approximately 1–2 km SE of 3M.

Previous studies along a distance gradient from the fluorochemical manufacturing plant (3M) in Antwerp have reported some of the highest recorded concentrations of PFAS in the plasma of wild birds residing near this facility [38,39] and, therefore, the choice of locations was based on previous monitoring studies [3,39,40]. To ensure coverage of the entire site, nest boxes for great tits were spread throughout the accessible parts of the areas and had a comparable habitat quality. Consideration was given to the fact that nest boxes will not get occupied when placed too close together. The number of nest boxes per location is, therefore, the result of making the most optimal use of the available accessible space in each location. Furthermore, efforts were made to maintain a comparable density of nest boxes across all areas, and the different microhabitats within each area were taken into account. Great tits are a model species used in ecotoxicological field studies because they breed and sleep in man-made nesting boxes, are often found in and around urban areas, and are known to reproduce in polluted areas [41,42]. Because of this, the sampling of these birds is relatively easy. Additionally, passerine bird species, such as great tits, have relatively small home ranges [36], which makes them suitable to monitor local contamination [37]. At Blokkersdijk/3M and Vlietbos, 38 and 19 nest boxes were placed, respectively. First, soil samples were continuously collected at 10 different depths (0–10 cm, 10–20 cm, 20–30 cm, 30–40 cm, 40–50 cm, 50–60 cm, 60–70 cm, 70–80 cm, 80–90 cm, and 90–100 cm) using an Edelman auger, maximum 1 m next to each nest box. From these, only five different layers (i.e., 0–10 cm, 20–30 cm, 50–60 cm, 70–80 cm, and 90–100 cm) were used for further analysis, due to the large amount of samples and possible mixing in between layers due to the use of an Edelman auger. The layers will be referred to as layer 1, layer 3, layer 5, layer 7, and layer 10, respectively. Additionally, the total organic carbon (TOC) content was determined in topsoil using the loss-on-ignition method [43] and is provided in Table S1. Furthermore, in a radius of 5 m surrounding each nest box, the aboveground parts of stinging nettles (*Urtica dioica*) as well as different terrestrial invertebrates (isopods, earthworms, snails, slugs, and spiders—no further taxonomic identification was made) present were collected manually during winter. These invertebrates were selected due to the varying diets among different taxa. Stinging nettles were chosen, as they were one of the only green fresh plants present during the time of sampling and are a food source to many invertebrate taxa. Five individuals from each invertebrate group were collected per nest box, when available.

Correspondingly, the nest boxes were visited after sunset during the winter of 2021–2022 (November 2021 until February 2022). Roosting great tits were caught inside these nest boxes and were ringed first to be able to identify them later on in case they were recaptured. After this, the two outer tail feathers, recognised by their white border, were collected and stored in 50 mL polypropylene (PP) tubes in a dark place to avoid any damage due to UV radiation. Hereafter, a blood sample was taken from the brachial vein, and a maximum of 150 µL was collected using microhematocrit heparinised capillary tubes (Microvette CB 300, SARSTEDT AG & Co. KG, Nümbrecht, Germany). Blood samples were centrifuged (4 °C, 10 min, 9279.4× *g*, Eppendorf Centrifuge 5414R, Eppendorf AG, Hamburg, Germany) to separate the blood plasma from the blood cells. The blood plasma was then transferred into Eppendorf tubes and stored at −80 °C until further analysis.

### 2.2. PFAS Analysis

All samples were pretreated differently prior to extraction. For soil and nettle samples, 300 ± 100 mg (dry weight (DW) for soil, wet weight (WW) for nettles) of material was used for extraction, and for plasma, 10 µL was used. Furthermore, whole invertebrates and tail feathers were used. All samples were homogenised prior to extraction. To all samples, 10 ng of a mass-labelled perfluoroalkyl carboxylic acid (PFCA) and perfluoroalkyl sulfonic acid (PFSA) internal standard (MPFAC-MXA, Wellington Laboratories, Guelph, ON, Canada) was added. Then, 10 mL of methanol (MeOH; HPLC gradient grade, VWR International, Leuven, Belgium) was added to feather samples, and 10 mL of acetonitrile (ACN; HPLC gradient grade, Acros Organics BVBA, Geel, Belgium) was added to all other samples. Then, different extraction procedures were followed according to the matrix. For plasma and soil samples, the extraction procedure followed the protocol described by Groffen et al. [44], for feathers, the extraction procedure followed the protocol described by Groffen et al. [45], and for all other samples, the extraction procedure followed the protocol described by Powley et al. [46], both with some minor modifications. The total amount of analysed soil samples, plants, invertebrates, and songbird samples is listed in Table S2. After extraction, samples were analysed for 29 PFASs using UPLC-MS/MS (ACQUITY TQD, Waters, Milford, MA, USA) (Tables S3 and S4). All details on the different sample pretreatments, extraction procedures, and UPLC-MS/MS analysis and quantification are provided in the Supplementary Material.

#### Quality Control and Assurance

As instrumental blanks, 100% acetonitrile (ACN; HPLC gradient grade, Acros Organics, Geel, Belgium) was injected on a regular basis to limit cross-over contamination between injections. Per batch of 15–20 samples, 1 procedural blank (i.e., 10 mL of ACN for soil and biota samples, and 10 mL of MeOH (HPLC gradient grade, VWR International, Leuven, Belgium) for feather samples) was used to correct for possible contamination that occurred during the extraction and analysis. Contamination in the procedural blanks (i.e., the average concentration of the procedural blanks within a batch) was subtracted from concentrations in the samples of the same batch. Average concentrations of 5 procedural blanks per batch, detected in the blanks per matrix, are provided in Table S3. Ranges are given when matrices were analysed in multiple batches. The limits of quantification (LOQs) were determined in the matrix as the concentration corresponding to a signal-to-noise ratio of 10 and are provided in Table S4. The recoveries of the individual mass-labelled internal standards used are provided in Table S5.

### 2.3. Statistical Analysis

The statistical analysis was performed in R Studio (Version 2022.07.02+576). The significance level for all tests was set at *p* ≤ 0.05. Data that were < LOQ were given imputed values based on a maximum likelihood estimation (MLE) to minimise left-skewness of the dataset [47]. PFAS concentrations that were below the LOQ in every sample were excluded from the tables, and only compounds with a detection frequency of ≥ 50% were included in the statistical analysis. Data normality was tested using the Shapiro–Wilk test, and standard deviations are presented in Table S20. Differences in concentrations between soil layers and between different terrestrial invertebrates were determined using ANOVAs (or their non-parametric equivalent if data were not normally distributed). Differences between terrestrial invertebrates were only analysed for Blokkersdijk/3M due to the higher abundance of compounds and higher detection frequencies compared to Vlietbos. Differences between soil layers were only determined when a compound was detected, with more than 50% detection frequency, in at least 3 out of 5 layers, and were determined for both sites separately. Afterwards, Tukey’s post hoc analysis was performed when significant differences were obtained. Associations between matrices (between PFASs in soil and PFASs in nettles or invertebrates, between PFASs in nettles and PFASs in invertebrates, or between PFASs in feathers and PFASs in plasma). Pearson correlations were used when data were normally distributed before or after log-transformation, while Spearman correlations were performed when data were not normally distributed, even after log-transformation. The used tests with their corresponding data can be found in Tables S21–S28. Associations were made for both sites individually and together, and were performed on data of birds or nestbox locations from which both matrices of the concentrations were > LOQ. Furthermore, differences between plasma and feather concentrations per site were assessed using paired t-tests. At last, the PFAS profiles and percentages were determined by correcting the PFAS concentrations with the molar masses of the respective PFASs and were descriptively compared among matrices. PFAS profiles were corrected for their corresponding molar masses due to the large differences between them, which provides a more accurate representation of the contribution of each PFAS to the profile, as short-chain PFASs would otherwise be underestimated.

## 3. Results

### 3.1. Soil PFAS Profiles and Concentrations

**Figure 2:**
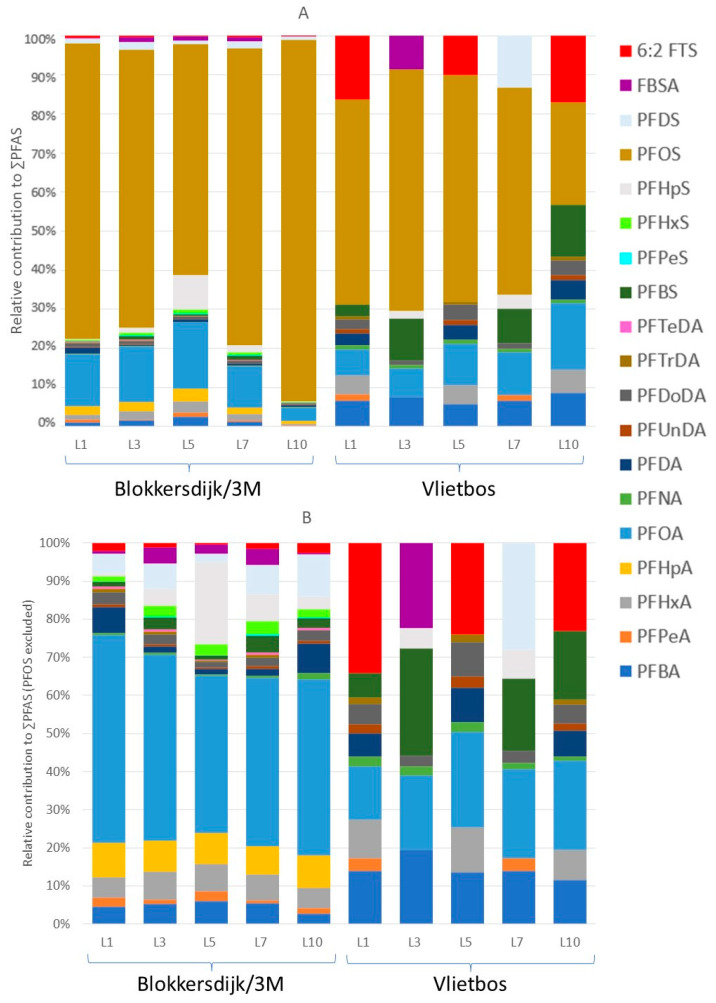
PFAS profiles of the different soil layers collected at both sampling sites based on concentrations in nmol/g DW. (**A**) PFAS profile for the different soil layers with PFOS included, and (**B**) PFAS profile for the different soil layers with PFOS excluded (scale is set to 100%, but it is not the real percentage). L1 = soil layer 0–10 cm deep, L3 = soil layer 20–30 cm deep, L5 = soil layer 40–50 cm deep, L7 = soil layer 60–70 cm deep, and L10 = soil layer 90–100 cm deep. All PFAS profiles were corrected for molar masses of the respective PFASs.

Median and mean PFAS concentrations, ranges, and detection frequencies in all soil layers analysed can be found in Tables S6–S10, and the mean concentrations corrected for the molar masses of the respective PFASs can be found in Table S19. Overall, the profiles of all five soil layers were dominated by perfluorooctane sulfonic acid (PFOS) at both Blokkersdijk/3M and Vlietbos (Figure 2A).

The profile of the deepest soil layer (L10) at Blokkersdijk/3M showed the highest contribution of PFOS (92%) with a concentration of 2679 ng/g DW, while at Vlietbos the deepest soil layer showed the lowest contribution for PFOS (26%), with a concentration of 2.91 ng/g DW. Without considering PFOS, both sampling sites had a different soil PFAS profile, where perfluorooctanoic acid (PFOA), perfluoroheptanoic acid (PFHpA), and perfluorohexanoic acid (PFHxA) showed the highest contributions at Blokkersdijk/3M (Figure 2B). Contrarily, at Vlietbos, the profile consisted, besides PFOS, mainly of perfluorobutanoic acid (PFBA), PFOA, perfluorobutane sulfonic acid (PFBS), and 6:2 fluorotelomer sulfonic acid (6:2 FTS). Strong differences in concentrations were observed between the two sites, with the mean ∑PFAS of the topsoil layer at Blokkersdijk/3M (1064 ng/g DW) being more than 50 times higher than that at Vlietbos (15.8 ng/g DW).

### 3.2. Vertical Distribution of PFASs in Soil

**Figure 3:**
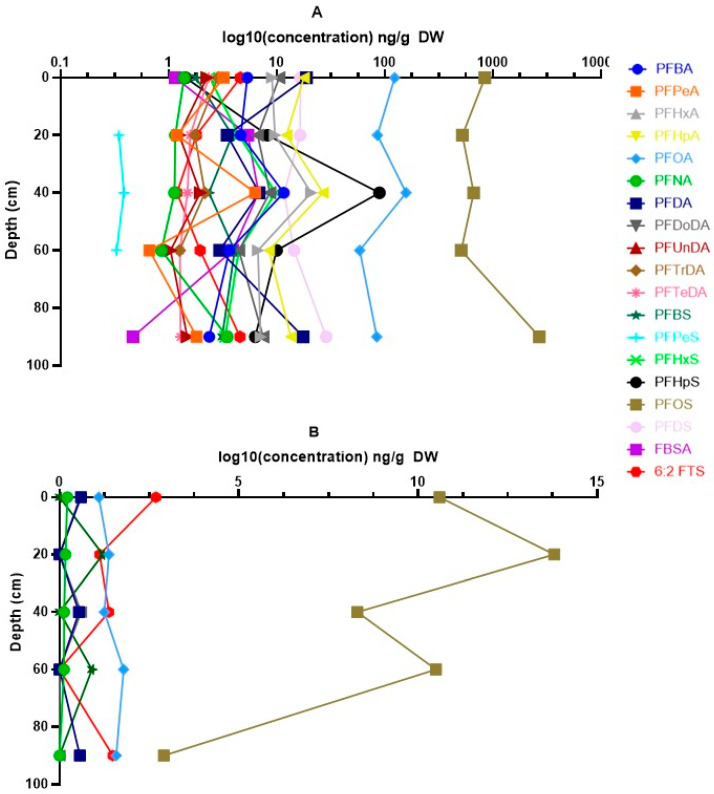
Vertical distribution of PFASs in the different soil layers analysed. (**A**) Depth profile of PFAS concentrations in soil at Blokkersdijk/3M. (**B**) Depth profile of PFAS concentrations in soil at Vlietbos. Concentrations are log10 transformed (ng/g DW) and depth is shown in cm. Error bars are omitted due to aesthetic reasons, but the variation in PFAS concentrations is shown in Tables S6–S10.

The vertical distribution of PFASs in the different soil layers for both sites is shown in Figure 3. At Blokkersdijk/3M, differences in concentrations among the different soil layers could be determined for PFBA, PFOA, perfluorononanoic acid (PFNA), perfluorodecanoic acid (PFDA), perfluoroundecanoic acid (PFUnDA), perflurododecanoic acid (PFDoDA), perfluorohexane sulfonic acid (PFHxS), and PFOS. Significant differences among layers were found for PFDA, PFUnDA, PFDoDA, PFBS, and PFHxS (*p* < 0.05). PFDA concentrations were significantly higher in the topsoil, layer 5, and layer 10 compared to layers 3 and 7. Similarly, the PFUnDA and PFDoDA concentrations were higher in the topsoil and layer 5 compared to layers 3 (only for PFUnDA) and 7. The opposite, with higher concentrations in layers 3 and 7 compared to the topsoil, layer 5, and layer 10, was observed for PFBS. For PFHxS, similar results were found as for PFBS, with higher concentrations in layers 3 and 7 compared to topsoil and layer 10, but lower concentrations were found in layer 7 compared to layer 5. At Vlietbos, only for PFOS significant differences were found among the different soil layers (*p* < 0.05). Here, layer 10 was significantly lower than all other soil layers analysed, and PFOS concentrations in layer 3 were significantly higher than in layer 5. For multiple compounds, i.e., perfluoropentanoic acid (PFPeA), PFHxA, PFHpA, PFTrDA, PFTeDA, PFBS, perfluoroheptane sulfonic acid (PFHpS), perfluorodecane sulfonic acid (PFDS), perfluorobutane sulfonamide (FBSA), and 6:2 FTS, not enough data were available to perform statistics, but for short-chain PFCAs (i.e., PFPeA, PFHxA, and PFHpA), the highest concentrations were often seen in either layer 3 or layer 5. The same pattern was noticed for FBSA, whilst 6:2 FTS was most present in both the topsoil and the deepest soil layer.

### 3.3. PFAS Profiles and Concentrations in Nettles

**Figure 4:**
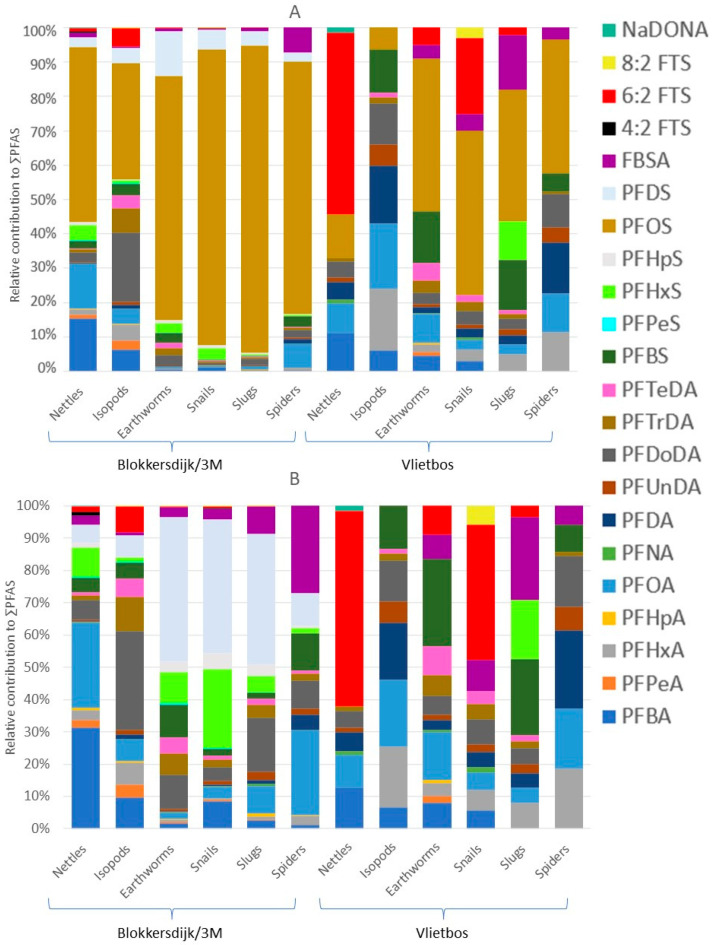
PFAS profiles of the different invertebrates and nettles collected at both sampling sites based on concentrations in nmol/g. (**A**) PFAS profiles of invertebrates and nettles sampled. (**B**) PFAS profiles of invertebrates and nettles sampled with PFOS excluded (scale is set to 100% but is not the real percentage). All PFAS profiles were corrected for the molar masses of the respective PFASs.

Median and mean PFAS concentrations, ranges, and detection frequencies in nettles can be found in Table S11. Compared to the soil profile, the nettles showed some minor differences, with short-chained PFASs and precursors having a larger contribution to the ∑PFAS in nettles (Figure 4A). However, similar to soil, the PFAS profile of the nettles was dominated by PFOS, with mean concentrations of 161 ng/g WW and 0.992 ng/g WW and contributions of 51% and 12% at Blokkersdijk/3M and Vlietbos, respectively. Different accumulation profiles were detected between sites, with PFBA (15%) and PFOA (12%) being the largest contributors to the profile besides PFOS at Blokkersdijk/3M (Figure 4B). At Vlietbos, 6:2 FTS was the most dominant in nettles, with a contribution of 52% and a mean concentration of 3.55 ng/g WW (Figure 4A).

### 3.4. PFAS Profiles, Concentrations, and Taxon-Specific Differences in Terrestrial Invertebrates

**Figure 5:**
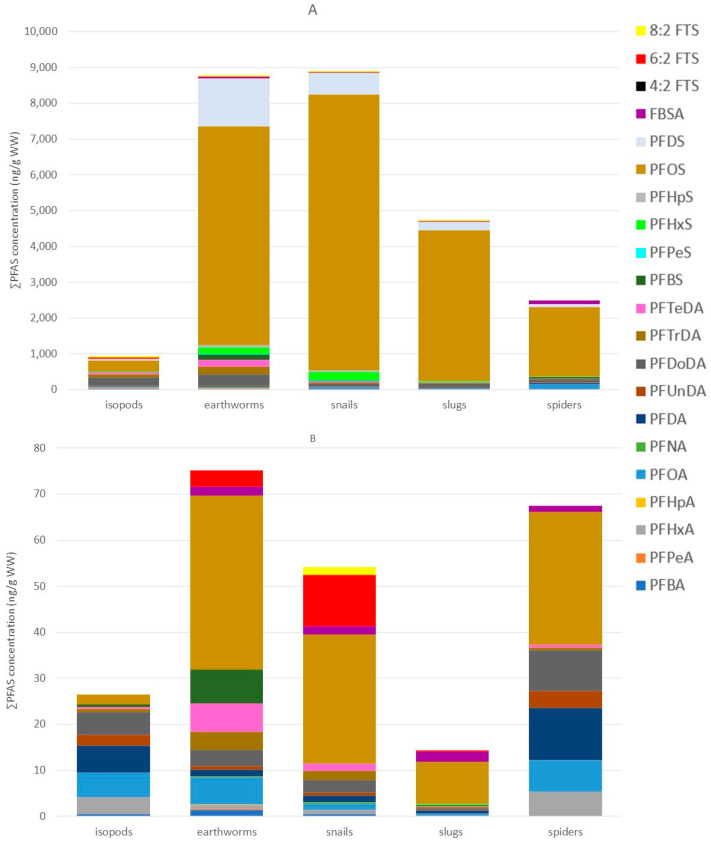
PFAS concentrations of the terrestrial invertebrates sampled at both sampling locations. (**A**): PFAS concentrations (ng/g WW) measured in the terrestrial invertebrates (isopods, earthworms, snails, slugs and spiders) at Blokkersdijk/3M from November 2021–February 2022 (**B**): PFAS concentrations (ng/g WW) measured in the terrestrial invertebrates (isopods, earthworms, snails, slugs and spiders) sampled at Vlietbos from November 2021–February 2022.

Median and mean PFAS concentrations, ranges, and detection frequencies of the terrestrial invertebrates can be found in Tables S12–S16. In terrestrial invertebrates, PFOS was overall the most dominant compound, except for isopods at Vlietbos, where PFOA (19%) showed the highest contribution, followed by PFDA (17%), PFBS (13%), and PFDoDA (12%; Figure 4 and Table S19). Mean PFOS concentrations found at Blokkersdijk/3M in isopods, earthworms, snails, slugs, and spiders were 313 ng/g WW, 6098 ng/g WW, 7687 ng/g WW, 4197 ng/g WW, and 1927 ng/g WW, respectively. At Vlietbos, mean PFOS concentrations were lower, with mean PFOS concentrations of 2.11 ng/g WW, 37.8 ng/g WW, 28.2 ng/g WW, 9.12 ng/g WW, and 28.8 ng/g WW for the same taxa, respectively. Besides the dominant compound PFOS, terrestrial invertebrates accumulated mainly long-chain PFASs, with much lower contributions for short-chain PFASs and precursor compounds (Figure 4B). At Blokkersdijk/3M, PFOA, PFDoDA, PFHxS, PFDS, and FBSA were, besides PFOS, the most dominant PFASs detected in terrestrial invertebrates, while at Vlietbos the profiles of the invertebrates showed more differences amongst each other. Notably, the smallest number of compounds were detected in the slugs at Vlietbos, while at Blokkersdijk/3M the slugs showed a more diverse accumulation profile. Then, at Blokkersdijk/3M, the highest total PFAS concentration was detected in snails, followed by earthworms > slugs > spiders > isopods, with mean ∑PFAS of 8858 ng/g WW, 8753 ng/g WW, 4717 ng/g WW, 2499 ng/g WW, and 914 ng/g WW, respectively (Figure 5A). At Vlietbos, earthworms showed the highest accumulation of PFASs, followed by spiders > snails > isopods > slugs, with mean ∑PFAS of 73.2 ng/g WW, 66.7 ng/g WW, 52.2 ng/g WW, 24.9 ng/g WW, and 11.3 ng/g WW, respectively (Figure 5B).

Further, at Blokkersdijk/3M, significant taxon-specific differences in concentrations were found for almost all compounds analysed: PFBA, PFHxA, PFOA, PFNA, PFDA, PFDoDA, perfluorotridecanoic acid (PFTrDA), perfluorotetradecanoic acid (PFTeDA), PFBS, PFHpS, PFOS, PFDS, and FBSA (*p* < 0.05; Figure 5A). Only for PFUnDA, no significant difference was found among taxa. Firstly, spiders contained significantly higher concentrations of PFBA compared to isopods, earthworms, and snails, while snails contained significantly higher PFBA concentrations compared to slugs. Secondly, isopods showed significantly higher levels of PFHxA compared to slugs, snails, and spiders. Furthermore, spiders contained significantly higher concentrations of PFOA, but significantly lower concentrations of PFNA and PFDA, compared to all other invertebrates analysed. Additionally, between isopods and earthworms, significant differences were also found for PFNA and PFDA, with higher PFNA levels in earthworms and higher PFDA levels in isopods. Further, earthworms showed significantly higher levels of PFDoDA compared to spiders, and higher PFTrDA levels compared to both spiders and slugs. Moreover, isopods contained significantly higher levels of PFTrDA compared to spiders. Subsequently, earthworms showed significantly higher PFTeDA and PFBS concentrations compared to all other invertebrates. Isopods had significantly lower levels of PFHpS and PFOS compared to earthworms, snails, and slugs, while earthworms contained significantly higher concentrations of PFHpS compared to spiders. In addition, isopods showed lower PFDS concentrations compared to earthworms and slugs. Earthworms contained significantly higher PFDS concentrations compared to spiders, and isopods showed significantly lower FBSA levels compared to all other invertebrates. For some compounds (i.e., PFPea, PFHpA, PFPeS, PFHxS, 6:2 FTS, and 8:2 FTS), statistical analysis could not be performed due to their lower detection frequencies. Here, the PFPeA concentrations were highest in isopods and earthworms, whereas they were scarcely detected in snails, slugs, and spiders. In contrast, PFHpA exhibited the highest detection frequencies in slugs, while it was rarely detected in other invertebrates. For PFSAs, PFPeS and PFHxS appeared to behave in a similar way, with the highest concentrations and detection frequencies observed in earthworms, snails, and slugs, while they were either not detected or only rarely detected in isopods and spiders. Furthermore, the fluorotelomer compounds (6:2 FTS and 8:2 FTS) were never detected in any spider samples, and 8:2 FTS was also absent in isopods, but traces of these compounds were found in the other invertebrates.

### 3.5. PFAS Profiles, Concentrations, and Matrix-Dependent Differences and Similarities in Great Tit Plasma and Feathers

**Figure 6:**
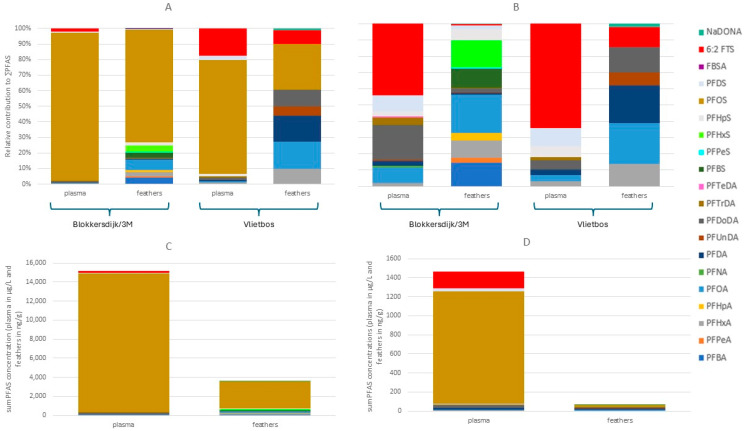
PFAS profiles and concentrations of the plasma and feathers of the great tits sampled at both sampling locations. (**A**) PFAS profiles of plasma and feathers of great tits based on concentrations in nmol/g. (**B**) PFAS profiles of plasma and feathers of great tits, with PFOS excluded (scale is set to 100% but is not the real percentage), based on concentrations in nmol/g. (**C**) PFAS concentrations of plasma and feathers sampled at Blokkersdijk/3M. (**D**) PFAS concentrations of plasma and feathers sampled at Vlietbos.

Median and mean PFAS concentrations, ranges, and detection frequencies measured in plasma and feathers of great tits can be found in Tables S17 and S19. Following the same trend as all other matrices analysed, the blood plasma and feathers of great tits showed a dominance of PFOS at both sampling sites (Figure 6A). Besides PFOS (95% at Blokkersdijk/3M and 73% at Vlietbos), 6:2 FTS was one of the dominant compounds found in the plasma at both Blokkersdijk/3M (2.2%) and Vlietbos (8.7%; Figure 6B). In the plasma and feathers sampled at Blokkersdijk/3M, more individual PFASs were detected compared to Vlietbos, with 12 and 20 PFASs detected in plasma and feathers of Blokkersdijk/3M, respectively, whilst in Vlietbos only 9 and 8 PFASs were detected in plasma and feathers, respectively. Furthermore, ∑PFAS concentrations were higher in both matrices at Blokkersdijk/3M, with mean ∑PFAS concentrations of 15,145 µg/L and 1409 µg/L in plasma (Figure 6C,D), and 3604 ng/g and 65.5 ng/g in feathers (Figure 6C,D) from birds at Blokkersdijk/3M and Vlietbos, respectively. As seen in other matrices, 6:2 FTS was more abundant at Vlietbos, compared to Blokkersdijk/3M, in both plasma and feathers. Contrarily to plasma, the PFAS profile of the feathers consisted of more compounds with, besides PFOS (72% in Blokkersdijk/3M and 29% in Vlietbos), a clear dominance of PFOA (6.5%), PFHxS (4.5%), PFBA (4.0%), PFBS (3.0%), and PFHxA (3.0%) at Blokkersdijk/3M, and PFOA (18%), PFDA (17%), PFDoDA (10%), PFHxA (9.6%), 6:2 FTS (8.7%), and PFUnDA (5.7%) at Vlietbos. Thus, distinct differences in the accumulation profiles were seen between plasma and feathers of birds in these sites. This was also observed in the concentrations, where, at Blokkersdijk/3M, significant differences between plasma and feathers were found for all compounds tested, i.e., PFOA, PFDA, PFDoDA, and PFOS (*p* < 0.05). At Vlietbos, no significant difference was found between plasma and feather concentrations for PFOA and PFDA, but sample sizes were smaller.

### 3.6. Associations Among Terrestrial Matrices

Associations between PFASs in soil and PFASs in biota, among biota, and between feathers and plasma of great tits were assessed. Associations were made for each site separately and for both sites combined. All exact *p*-values and corresponding correlation coefficients can be found in Tables S21–S28.

Firstly, between PFASs in soil and PFASs in nettles at Blokkersdijk/3M, significant positive correlations were found for all long-chained PFCAs analysed and PFOS, while at Vlietbos, only for PFOS was a significant positive correlation found. When combining data from both sites together, again for all long-chained PFCAs and PFOS, significant positive correlations (*p* < 0.05) were found (Table S21). Furthermore, between soil and terrestrial invertebrates, all significant correlations were positive, and mainly for long-chained PFCAs and PFOS (Table S22). Between PFASs in soil and PFASs in isopods and earthworms, significant positive correlations (*p* < 0.05) were seen for almost all long-chained PFCAs and PFOS, but also for PFPeA and PFHxA, both short-chained PFCAs. Between PFASs in soil and PFASs in snails, significant positive associations were found for long-chained PFCAs, PFOS, and PFBA. Between PFASs in soil and PFASs in slugs, the same trend was seen with associations for long-chained PFCAs, PFOS, and PFHxA. Finally, between PFASs in soil and PFASs in spiders, only for PFOS and PFOA were significant associations found (*p* < 0.05). Then, between PFASs in soil and PFASs in plasma, only one positive significant association was found for PFDoDA at Blokkersdijk/3M (r(17) = 0.51, *p* = 0.026) and one significant positive association for PFOA at Vlietbos (r(12) = 0.64, *p* = 0.014; Table S23). When considering both sites together, significant positive associations were found for PFOA (r(31) = 0.54, *p* = 0.001) and PFDA (r(32) = 0.48, *p* = 0.004). Between PFASs in soil and PFASs in feathers at Blokkersdijk/3M, significant positive associations were found for PFDoDA (r(16) = 0.66, *p* = 0.003) and PFOS (r(16) = 0.62, *p* = 0.006), whilst at Vlietbos no significant associations were found. When considering both sites, both for PFOA (r(30) = 0.61, *p* = 0.0002) and PFDoDA (r(30) = 0.47, *p* = 0.006), significant positive associations were found between soil and feathers, whilst for PFOS a significant negative association was found (r(31) = −0.49, *p* = 0.004; Table S23).

Furthermore, between PFASs in nettles and PFASs in terrestrial invertebrates, all significant associations found were positive, and mainly for long-chained PFCAs and PFOS (Table S24). Then, between PFASs in nettles and PFASs in plasma of great tits, no significant associations were found for the sites individually (Table S25). When combining data from both sites, significant positive associations between PFASs in nettles and PFASs in plasma were found for PFOA (r(36) = 0.48, *p* = 0.002) and PFDA (r(34) = 0.42, *p* = 0.013). Between PFASs in nettles and PFASs in feathers of great tits, only one significant positive association was found for PFDA (r(19) = 0.47, *p* = 0.030) at Blokkersdijk/3M, and at Vlietbos no significant associations were found. When combining data from both sites, only for PFOA a positive significant association was found (r(36) = 0.49, *p* = 0.002). Further, between PFASs in terrestrial invertebrates and PFASs in plasma, only a very limited number of significant associations were found (Table S26). Most significant associations were found between PFASs in earthworms and PFASs in plasma, where at Blokkersdijk/3M significant positive associations were detected for long-chained PFASs (PFOA, PFDA, and PFTrDA), but at Vlietbos no significant associations were found. When combining data from both sites, significant positive associations were found for PFOA (r(32) = 0.47, *p* = 0.005) and PFDA (r(31) = 0.51, *p* = 0.002). Furthermore, only sporadic significant associations were observed between PFASs in isopods, snails, and slugs and PFASs in plasma, mostly for either PFOA, PFDA, and/or PFOS. At last, between PFASs in spiders and PFASs in plasma, no significant associations were found. Furthermore, also between PFASs in terrestrial invertebrates and the feathers of great tits, a limited number of significant associations were found (Table S27). Here, most significant associations with feathers were found for snails, with significant positive associations at Blokkersdijk/3M for PFOA (r(12) = 0.56, *p* = 0.037) and PFDoDA (r(10) = 0.79, *p* = 0.002), and for PFDA (r(5) = −0.94, *p* = 0.00) at Vlietbos. For both sites together, significant associations between PFASs in snails and feathers were seen for PFOA (r(18) = 0.56, *p* = 0.008) and PFOS (r(19) = 0.69, *p* = 0.0006). Then, between PFASs in isopods and earthworms and PFASs in feathers, significant positive associations were mostly found for either PFOA or PFDoDA (*p* < 0.05). Finally, between PFASs in slugs and PFASs in feathers, only for PFOS were significant associations found for both sites together (r(26) = 0.77, *p* = 1.96 × 10^−6^). The same was seen for PFASs in spiders and PFASs in feathers (r(16) = 0.60, *p* = 0.008).

Finally, between PFASs in plasma and PFASs in feathers, only for PFOS was a significant positive association found between plasma and feather concentrations at Blokkersdijk/3M (r(16) = 0.63, *p* = 0.005). For all other compounds tested, no significant associations were found between plasma and feathers (*p* > 0.05, Table S28). When grouping both sites, both for PFOA (r(24) = 0.40, *p* = 0.045) and PFOS (r(21) = 0.61, *p* = 0.002), significant positive correlations were found between plasma and feather concentrations.

## 4. Discussion

### 4.1. Soil PFAS Profiles and Concentrations

The dominance of PFOS in the soil samples taken at both sampling sites can be explained by the large production volumes and discharge of this compound by the fluorochemical facility before the phase-out in 2002. Nonetheless, the published PFOS discharge was still 10.1 kg/year in 2021 [48]. Additionally, several short-chain compounds were detected within the soil, which characterises the shift to the use of shorter-chained compounds in recent years. The different soil PFAS profile at Vlietbos compared to Blokkersdijk/3M (e.g., higher presence of 6:2 FTS at Vlietbos) may indicate other sources of PFASs in the vicinity of this sampling location, in addition to the fluorochemical facility. Short-chain PFCAs (e.g., PFBA, PFPeA, PFHxA, and PFHpA), which are known degradation products of several precursors [49], including 6:2 FTS, which was frequently detected in soil at Vlietbos, were scarcely detected in soil and other matrices. This suggests limited (bio)transformation of this compound in the area. Additionally, the present study found clear differences in the magnitudes of soil PFAS concentrations between both sampling sites, which may be explained by the distance between the sampling sites and the fluorochemical point source. The sampling location Blokkersdijk/3M consisted partially of the site of the fluorochemical facility itself, together with the adjacent nature reserve Blokkersdijk, east from the facility. Since the dominant wind direction in Belgium comes from the southwest [50], the northeast should be affected mostly through any possible aerial deposition occurring near the facility. Vlietbos, on the other hand, is located 1–2 km in the southeast direction of this fluorochemical facility, which explains the lower concentrations measured at Vlietbos due to less aerial deposition, compared to Blokkersdijk/3M. Furthermore, the PFOS and PFOA concentrations found at Vlietbos in the present study were in the same order of magnitude as previous studies performed in this area [14,16,28]. However, comparison with previous studies conducted in Blokkersdijk and 3M was not possible, as the present study treated these locations as one site, whereas earlier studies analysed them separately.

### 4.2. Vertical Distribution of PFASs in Soil

Some clear patterns regarding the vertical distribution of PFASs in soil were found in the present study. Most long-chain PFASs (i.e., PFOA, PFDA, PFDoDA, PFTrDA, and PFTeDA) had higher concentrations in topsoil layers compared to deeper layers, whilst for PFOS and PFDS, the highest concentrations were measured in the deepest soil layer. Additionally, PFDA, PFUnDA, and PFDoDA were higher in layers 1, 5, and 10, whilst PFBS and PFHxS concentrations were higher in soil layers 3 and 7. These results indicate differences due to the functional group of these compounds, i.e., differences between PFCAs and PFSAs. Such differences between functional groups were also seen in the study of Gan et al. [22], where topsoil concentrations of PFOA were higher than in other soil layers. Additionally, PFBS and PFHxS concentrations in the present study followed the same trend as PFOS and PFDS, with concentrations in the topsoil being lower compared to deeper layers. The study of Zhou et al. [23] found that mean concentrations of both PFBS and PFHxS were higher in middle soil layers (10–20 cm) compared to the topsoil layer (0–10 cm), but the lowest concentrations were seen in the deepest layer (20–30 cm). Our results are contradictory to earlier findings where PFSAs showed stronger sorption capacities to soil compared to PFCAs [51]. PFSAs should, therefore, bind more effectively to the topsoil layer compared to PFCAs, which is not the case in the present study. Additionally, it is unclear why some compounds are more abundant in layers 1, 5, and 10, whilst others have higher concentrations in layers 3 and 7. These contradictory results may be explained by differences in soil physicochemical properties (e.g., total organic carbon content, particle size, moisture content, pH, etc.) in between soil layers, which might result in different affinities of some PFASs for certain soil layers [14]. Unfortunately, the present study did not investigate the soil characteristics, so future studies would be necessary to investigate the sorption and leaching of PFASs across different soil layers in this area. Additionally, the interactions between these different soil characteristics may be more important in the process of sorption and leaching of PFASs to soil, as opposed to the chemical structure of the PFASs [14,52,53]. Finally, the timing of the production and the release of other compounds in the environment may also have an impact on the distribution of PFASs in the different soil layers.

### 4.3. PFAS Profiles and Concentrations in Nettles

The profile of the nettles was consistent with the soil PFAS profile at both sites, with some minor differences. Interestingly, the composition profile of the nettles was predominantly characterised by long-chain PFASs and 6:2 FTS, with a relatively smaller proportion of short-chain PFASs (PFBA and PFHxA). This was unexpected, as the uptake of PFASs in plants is typically dependent on the chain length of the compounds [54], where shorter-chained compounds are known to be more readily absorbed by plant roots due to their smaller molecular sizes [55]. On the other hand, the soil characteristics and resulting bioavailability and/or potential to leach also affect the uptake. The presence of long-chain PFASs (e.g., PFOS) in the aboveground parts of these nettles may be attributed to the significantly high levels of these compounds in the soil, potentially making them more bioavailable for plant uptake compared to other cases. However, this hypothesis requires further investigation. Groffen et al. [16] also analysed nettles at Vlietbos for PFASs and observed similar concentrations for some compounds (PFDA and PFOS), while for other compounds (i.e., PFBA, PFOA, and PFBS), the concentrations in nettles were even higher than those reported in our study. Furthermore, the present study was also able to detect a mean 6:2 FTS concentration of 3.55 ng/g WW, a compound which the previous study did not include in their analysis. Not much is known yet regarding the accumulation of precursors in plants, but Che et al. [56] did see accumulation of precursor compounds (including 6:2 FTS) in the roots, stems, and leaves of cabbage plants. Additionally, Rijnders et al. [7] analysed nettles in Vlietbos and 3M and found similar concentrations in the aboveground parts of nettles for PFBA, PFDA, and PFUnDA, but PFAS concentrations in nettles at Blokkersdijk/3M of the present study were often higher (PFBA, PFHxA, PFOA, PFNA, PFDA, PFUnDA, PFDoDA, and PFOS), except for PFBS, which had a lower mean value in the present study. Differences between these field studies may be attributed to variations in sampling techniques, as well as the specific locations from which the samples were collected. The distribution of PFASs in the soil near this facility is highly uneven (Tables S6–S10 [14,28]) and, when combined with varying bioavailability influenced by factors such as soil characteristics (e.g., clay content and organic matter) [16], weather conditions, and other uncontrollable variables, this can lead to a range of PFAS concentrations accumulating in the plants present. Nevertheless, field studies examining PFASs in terrestrial plants are crucial for understanding the behaviour of these compounds in the field, as well as their potential uptake by animals and humans. Furthermore, additional research is needed to investigate the accumulation of PFASs in other terrestrial non-crop species.

### 4.4. PFAS Profiles, Concentrations, and Taxon-Specific PFAS Accumulation in Terrestrial Invertebrates

Overall, the accumulation profile within the terrestrial invertebrates consisted mainly of long-chained PFASs, even when PFOS was excluded, and was expected since long-chained PFASs are known to mainly accumulate within animals due to their proteinophilic character, higher hydrophobicity, and longer half-lives compared to their shorter-chained homologues [57]. Besides this, isopods contained the highest ∑short-chained PFASs of all invertebrates, which is most likely related to their detritivorous diet. Contrarily to isopods, snails and slugs accumulated lower concentrations of shorter-chained compounds, which might be due to their omnivorous diet, where the PFAS profile in snails might be similar to those of their animal prey, but this hypothesis requires further investigation. Additionally, the present study mainly detected long-chain PFASs in nettles, which might be another explanation since snails prefer to eat on fresh plants too [58]. Surprisingly, no fluorotelomer compounds were found in spiders, while traces of these were found in all other invertebrates. To the best of our knowledge, no other field studies on PFAS accumulation in terrestrial invertebrates have included such compounds. These differences in accumulation profiles among the different terrestrial invertebrates are most likely reflecting their corresponding diets.

Among the studied taxa, we had expected that PFAS concentrations would be highest in spiders, due to their higher trophic position relative to the other invertebrates. Nonetheless, at Blokkersdijk/3M, the ∑PFAS concentration was highest in snails, and at Vlietbos in earthworms. These higher concentrations in both taxa may be the result that both taxa were collected from the ground, where they were in close contact with the contaminated soil. Isopods, on the other hand, were also collected on the ground, but accumulated the lowest ∑PFAS concentrations of all invertebrates, which might be linked to their frequent moulting process [59]. Such differences in concentrations among terrestrial invertebrates have been reported before [16], and were most likely linked to differences in diets between these taxa, but other influencing factors may also have played a role in the accumulation rate (e.g., metabolic activity of invertebrates and environmental conditions).

An earlier study performed at Vlietbos found mean PFBA, PFOA, and PFOS concentrations of 0.692 ng/g WW, 4.20 ng/g WW, and 1.20 ng/g WW, respectively, in isopods, which lays in the same range as the findings of the present study. However, additional compounds were identified in the isopods at Vlietbos (i.e., PFHxA, PFDA, PFUnDA, PFDoDA, and PFTrDA). These compounds were also included in the analysis of the study of Groffen et al. [28]; however, they exhibited higher LOQs (excluding PFDoDA) compared to the present study, which may explain the higher number of detections observed herein. Groffen et al. [16] analysed isopods and spiders from Vlietbos, and concentrations of the compounds detected in both studies were of similar orders of magnitude; however, the present study identified additional compounds in both species (i.e., PFHxA, PFUnDA, PFDoDA, and PFTrDA in isopods and PFHxA, PFUnDA, PFDoDA, PFTrDA, and FBSA in spiders). Another study performed at Blokkersdijk found median PFOS concentrations of 497 ng/g WW, 3090 ng/g WW, and 2410 ng/g WW in isopods, slugs, and earthworms, respectively [29]. The PFOS concentrations found in the present study were similar to those in the study of D’Hollander et al. [29], except for the earthworms, where the present study found higher median concentrations. This might be due to the location of the sampling: the study of D’Hollander et al. [29] only sampled at Blokkersdijk for this specific site, while the present study also took additional samples on the fluorochemical plant site itself, which might explain the higher PFOS concentrations of the present study. Furthermore, Zhu and Kannan et al. [60] investigated PFCA concentrations in earthworms near a contaminated site in Washington County, Ohio, USA, and found 4–7 times higher PFOA, PFNA, PFDA, and PFUnDA concentrations compared to the present study, while soil concentrations of these compounds were only slightly higher compared to the present study. Considering that soil properties can influence the bioavailability of PFASs in soil, it is plausible that the compounds in the present study were less bioavailable, leading to lower concentrations. Nonetheless, the present study observed concentrations of PFHpA and PFDoDA in earthworms that were 2–4 times higher compared to those reported by Zhu and Kannan et al. [60], which is likely attributed to the higher soil concentrations of PFHpA and PFDoDA measured in the current study. This indicates differences in accumulation between hotspots, but also in the same hotspot areas. Distinct point sources exhibit unique contamination footprints, characterised by varying PFASs present at different concentrations. These concentrations also vary within the same hotspot area and may also differ from year to year. Therefore, continuous monitoring of PFAS concentrations across different steps of the terrestrial ecosystem is essential to evaluate temporal changes.

### 4.5. PFAS Profiles, Concentrations, and Matrix-Dependent Differences and Similarities in Great Tit Plasma and Feathers

When comparing our results to those of Groffen et al. [42], which were obtained in the same study area, the PFAS concentrations in both feather and plasma samples were lower in the present study, at both sites. This might be due to the phase-out of these compounds and/or the potential decrease in bioavailability of certain PFASs due to, e.g., leaching to deeper soil layers after the lowering of the production. Remarkably, the present study found higher PFDoDA concentrations in both plasma and feathers at both sampling sites, compared to the earlier study of Groffen et al. [42], which was unexpected since other studies reported decreases in plasma levels of animals and humans [61,62]. Furthermore, several additional PFASs were detected in the feathers at Blokkersdijk/3M, which were not detected (PFHpA) or not analysed (PFPeS, PFHpS, FBSA, 6:2 FTS, and NaDONA) in the study of Groffen et al. [42].

When comparing both matrices, the plasma and feathers of the great tits showed a very different accumulation profile. Compared to feathers, the profile of the plasma contained less compounds, which suggests that both matrices reflect other routes of exposure, have different affinities for certain PFASs, and/or that both matrices represent a different period of exposure. Earlier studies on micropollutants have suggested that blood plasma gives a representation of recent exposure through diet, while feathers reflect the long-term exposure, from moulting until the moment of sampling [42,63,64]. During formation and growth, feathers are connected to the bloodstream, and contaminants present in the blood may then be deposited into feathers [65]. Furthermore, feathers may have also been exposed in other ways compared to plasma, for example, during preening via preen oil, or through external sources (i.e., contact with polluted air, dust, and water) [37]. Therefore, the present study can conclude that feathers are not useful for internal PFAS monitoring, but they can give additional information on the environment to which the great tits were exposed (from moulting until sampling).

### 4.6. Associations Among Terrestrial Matrices

Between PFASs in soil and PFASs in the different biota, multiple significant associations were found in the present study. These significant associations indicate that some PFAS concentrations detected in both matrices were statistically related, which possibly suggests that (1) these PFASs in both matrices were originating from the same source, (2) there is a possible environmental transfer of PFASs between these matrices, (3) bioaccumulation, or even biomagnification, occurred from one matrix to another, and (4) some matrices may be used as a potential proxy for estimating PFAS concentrations in higher levels of the ecosystem.

Firstly, we found significant associations between PFAS concentrations in soil and nettles for several long-chained PFCAs and PFOS, but not for any short-chain PFASs. Additionally, the functional group seems to play a role since for PFCAs, only significant positive associations were found, whilst for PFOS a significant negative association was found. Here, the presence of long-chained PFASs in nettles is most likely due to the uptake from soil, whilst other PFASs in the nettles most likely originate from other sources (e.g., rainwater). Further, the present study found significant associations between long-chained PFASs in isopods and soil, which indicated that long-chain PFAS concentrations in isopods reflect those in soil, possibly suggesting that isopods accumulate long-chain PFASs by ingesting soil particles along their diet, something which was also observed by Groffen et al. [28]. The same trend was seen in the present study, where significant associations were found for both long- and short-chained PFASs between PFASs in soil and PFASs in earthworms, snails, and slugs. These results show the possibility of using soil to estimate the PFAS concentrations present in some terrestrial invertebrates, which was not seen for spiders since only a few significant associations were found between PFASs in soil and spiders. Spiders were most likely less exposed to PFASs through soil and have other more dominant routes of exposure (e.g., diet of other invertebrates). These results were expected, as isopods, earthworms, and slugs (and even snails) are in closer contact with soil compared to spiders and, therefore, soil was most likely a more important PFAS exposure pathway to these invertebrates than it was to spiders. Finally, between soil and great tit matrices, a lower amount of significant associations were found compared to the invertebrate matrices. Here, PFASs in feathers showed more associations with PFASs in soil, compared to PFASs in plasma, which might be attributed to the potential contamination from external sources (e.g., dust particles and soil particles), as discussed in Section 4.5. Nonetheless, some significant associations were found between PFASs in plasma and PFASs in soil, which indicates that some PFASs in the plasma were possibly originating from the same source as the PFASs detected in soil, and that soil might potentially be used as a proxy to estimate some PFAS concentrations in great tits.

Furthermore, between PFASs in nettles and terrestrial invertebrates, overall, the present study found significant associations for long-chained PFCAs and PFOS. These results were unexpected since plants are known to predominantly accumulate short-chain PFASs but may be explained by the high concentrations of long-chained PFCAs and PFOS found in nettles. Given these high concentrations in soil, it is plausible that the nettles accumulated higher concentrations of PFASs from this source compared to other cases. Between PFASs in nettles and great tits, few significant associations were found, which indicates that great tits were most likely not exposed to nettles, and might have different routes of exposure. Then, between PFASs in terrestrial invertebrates and PFASs in great tits, barely any significant associations were found, with an exception for long-chained PFASs in earthworms and plasma. Since terrestrial invertebrates (i.e., isopods, earthworms, snails, and slugs) are no direct food sources for great tits, it was expected that less significant associations were found. Here, we expected to find most associations between PFASs in spiders and great tits, but no associations were found between both matrices. Spiders are known food sources for great tits, but the great tits sampled during the winter might have fed on different food sources, resulting in no associations.

Finally, only a small number of associations were found between PFASs in plasma and PFASs in feathers, as found in the study of Groffen et al. [42]. Therefore, we agree with the statements of Jaspers et al. [37] that feathers cannot always be successfully used as monitors for environmental pollution. Here, feathers rather provide a snapshot of the pollutant load in the environment of the bird, more than reflecting internal concentrations in the bird [37], as often only for a limited number of compounds were significant associations found [37,42]. Additionally, at Blokkersdijk/3M, all compounds tested differed significantly in concentrations between plasma and feather samples. Since PFASs are proteinophilic [66], such differences in accumulation between both matrices may also be due to different proteins being present in both plasma and feathers, in combination with differences in protein binding affinity of different PFASs [67]. Contrarily, such differences were not seen at Vlietbos, which might be due to the lower sample sizes and lower detection frequencies of some compounds. At last, although there is no extensive knowledge on washing procedures for PFASs in feathers yet, a pretreatment, as suggested by Jaspers et al. [37] and Løseth et al. [68], was followed, which included the washing of the feathers with Milli-Q to remove dust and particles from the feathers before analysis, followed by a thorough wash using tweezers to be able to clean in between the barbs. Since not much is known regarding the external pollution, it might be that some hydrophobic PFASs remained stuck to these feathers, but this can, at present, not be confirmed. Hence, further studies are needed to determine the extent of this potential external contamination.

## 5. Conclusions

The present study found that the accumulation of PFASs in different terrestrial matrices showed many similarities, but also strong differences depending on the taxa. Additionally, there is still a need for more research regarding the accumulation of PFASs in different non-crop terrestrial plant species to further evaluate the potential exposure risk to other herbivorous biota. The present study was also able to determine some proxy matrices, which might be used in the future to establish predictive models, but more research from different locations is needed. Here, PFASs in soil showed statistically significant similarities to PFASs in nettles, isopods, earthworms, snails, and slugs, but not for spiders and great tit plasma or feathers. Furthermore, PFASs in nettles were associated with PFASs in isopods, earthworms, snails, and slugs, but less in spiders and barely in great tits. Additionally, only some PFASs in earthworms were associated with PFASs found in great tit plasma, but not for other terrestrial invertebrates. Finally, the present study confirmed that feathers are not suitable as a less-invasive biomonitoring matrix for internal plasma concentrations of PFASs in birds, except for PFOS. However, feathers can indicate the presence of PFASs in birds over a longer exposure time. Furthermore, there is still a need for studies evaluating the transfer and biomagnification of PFASs in terrestrial ecosystems by calculating different accumulation factors to quantify the bioaccumulation and biomagnification of these compounds, which should ideally be supported with stable isotope analysis.

## Data Availability

Raw data will be made available upon request.

## Supplementary Materials

The following supporting information can be downloaded at: https://www.mdpi.com/article/10.3390/toxics13080689/s1. Table S1: Total organic carbon (TOC) content per soil sampletaken at the corresponding nest box numbers. TOC values are given in percentages; Table S2: Number of analysed samples at both sampling locations; Table S3: mean concentrations of the blank samples per batch. The values that are shown are averages of 5 procedural blanks per batch; Table S4: Limit of quantification (LOQ) of all PFAS analysed in the different matrices evaluated: soil (ng/g DW), nettles (ng/g WW), invertebrates (ng/g WW), plasma (pg/µL) and feathers (ng/g WW); Table S5: percentages of the recoveries of the individual mass-labelled internal standards per matrix analysed in the study; Table S6: median and mean PFAS concentrations (ng/g DW), ranges and detection frequencies of the topsoil layer (0–10 cm), N = 18 Vlietbos, N = 33 Blokkersdijk/3M; Table S7: median and mean PFAS concentrations (ng/g DW), ranges and detection frequencies of soil layer 3 (20–30 cm), N = 18 Vlietbos, N = 35 Blokkersdijk/3M; Table S8: median and mean PFAS concentrations (ng/g DW), ranges and detection frequencies of soil layer 5 (40–50 cm), N = 17 Vlietbos, N = 35 Blokkersdijk/3M; Table S9: median and mean PFAS concentrations (ng/g DW), ranges and detection frequencies of soil layer 7 (60–70 cm), N = 18 Vlietbos, N = 33 Blokkersdijk/3M; Table S10: median and mean PFAS concentrations (ng/g DW), ranges and detection frequencies of soil layer 10 (90–100 cm), N = 18 Vlietbos, N = 29 Blokkersdijk/3M; Table S11: median and mean PFAS concentrations (ng/g WW), ranges and detection frequencies in the nettles sampled at Vlietbos (N = 18) and Blokkersdijk/3M (N = 35); Table S12: median and mean PFAS concentrations (ng/g DW), ranges and detection frequencies in the isopods sampled at Vlietbos (N = 16) and Blokkersdijk/3M (N = 36), each value was a mean value of 5 different isopods per nest box; Table S13: median and mean PFAS concentrations (ng/g WW), ranges and detection frequencies in the earthworms sampled at Vlietbos (N = 17) and Blokkersdijk/3M (N = 28), each value was a mean value of 5 different earthworms per nest box; Table S14: median and mean PFAS concentrations (ng/g WW), ranges and detection frequencies in the snails sampled at Vlietbos (N = 8) and Blokkersdijk/3M (N = 15), each value was a mean value of 5 different snails per nest box; Table S15: median and mean PFAS concentrations (ng/g WW), ranges and detection frequencies in the slugs sampled at Vlietbos (N = 14) and Blokkersdijk/3M (N = 18), each value was a mean value of 5 different slugs per nest box; Table S16: median and mean PFAS concentrations (ng/g WW), ranges and detection frequencies in the spiders sampled at Vlietbos (N = 6) and Blokkersdijk/3M (N = 11), each value was a mean value of 5 different spiders per nest box; Table S17: median and mean PFAS concentrations (µg/L), ranges and detection frequencies in the plasma of great tits sampled at Vlietbos (N = 15) and Blokkersdijk/3M (N = 23); Table S18: median and mean PFAS concentrations (ng/g), ranges and detection frequencies in the feathers sampled at Vlietbos (N = 15) and Blokkersdijk/3M (N = 23); Table S19: Mean PFAS concentrations found in each matrix at both sampling sites; Blokkersdijk/3M (BD3M) and Vlietbos (VB). Concentrations are expressed in nmol/g, consistent with the units used in the development of the PFAS profiles of each matrix; Table S20: Relative standard deviation (%) based on the mean concentrations found in each matrix. Table S21: Results of pearson (p) or spearman (s) correlations made between PFAS in soil and PFAS in nettles found at Blokkersdijk/3M (BD3M), Vlietbos (VB) and both sites combined (BD3M-VB), with significant correlations in bold; Table S22: Results of pearson (p) or spearman (s) correlations made between PFAS in soil and PFAS in terrestrial invertebrates found at Blokkersdijk/3M (BD3M), Vlietbos (VB) and both sites combined (BD3M-VB); Table S23: Results of pearson (p) or spearman (s) correlations made between PFAS in soil and PFAS in great tits sampled at Blokkersdijk/3M (BD3M), Vlietbos (VB) and both sites combined (BD3M-VB); Table S24: Results of pearson (p) or spearman (s) correlations made between PFAS in nettles and PFAS in terrestrial invertebrates found at Blokkersdijk/3M (BD3M), Vlietbos (VB) and both sites combined (BD3M-VB); Table S25: Results of pearson (p) or spearman (s) correlations made between PFAS in nettles and PFAS in great tits sampled at Blokkersdijk/3M (BD3M), Vlietbos (VB) and both sites combined (BD3M-VB); Table S26: Results of pearson (p) or spearman (s) correlations made between PFAS in terrestrial invertebrates and PFAS in great tit plasma sampled at Blokkersdijk/3M (BD3M), Vlietbos (VB) and both sites combined (BD3M-VB); Table S27: Results of pearson (p) or spearman (s) correlations made between PFAS in terrestrial invertebrates and PFAS in great tit feathers sampled at Blokkersdijk/3M (BD3M), Vlietbos (VB) and both sites combined (BD3M-VB); Table S28: Results of pearson (p) or spearman (s) correlations made between PFAS in plasma and PFAS in feathers of great tits sampled at Blokkersdijk/3M (BD3M), Vlietbos (VB) and both sites combined (BD3M-VB).

## Abbreviations

The following abbreviations are used in this manuscript:

PFASs	Per- and poly-fluoroalkyl substances
TOC	Total organic carbon
PP	Polypropylene
UPLC-MS/MS	Ultra-performance liquid chromatography coupled tandem mass spectrometry
ACN	Acetonitrile
LOQ	Limit of quantification
MLE	Maximum likelihood estimation
PFOS	Perfluorooctanesulfonic acid
PFOA	Perfluorooctanoic acid
PFHpA	Perfluoroheptanoic acid
PFHxA	Perfluorohexanoic acid
PFBA	Perfluorobutanoic acid
PFBS	Perfluorobutane sulfonic acid
FTS	Fluorotelomer sulfonic acid
PFNA	Perfluorononanoic acid
PFDA	Perfluorodecanoic acid
PFUnDA	Perfluoroundecanoic acid
PFDoDA	Perfluorododecanoic acid
PFHxS	Perfluorohexane sulfonic acid
PFPeA	Perfluoropentanoic acid
PFHpS	Perfluoroheptane sulfonic acid
PFDS	Perfluorodecane sulfonic acid
FBSA	Perfluorobutane sulfonamide
PFTrDA	Perfluorotridecanoic acid
PFTeDA	Perfluorotetradecanoic acid
PFCA	Perfluorocarboxylic acid
PFSA	Perfluorosulfonic acid
