# Supplementary material for: Distribution of Legacy and Emerging PFASs in a Terrestrial Ecosystem Located near a Fluorochemical Manufacturing Facility
Source: Toxics. 2025 Aug 19;13(8):689. doi: 10.3390/toxics13080689 (PMC12390023; doi:10.3390/toxics13080689)
Supplement: Supplementary file 1 [file toxics-13-00689-s001.zip › toxics-3787043-supplementary.pdf]

Supplementary Information

PFAS analysis: sample pretreatment

All samples were weighed prior to extraction, and soil samples were oven-dried at 60°C before weighing. Whole invertebrates and tail feathers were used, and feathers were washed thoroughly with Milli-Q water, as suggested by Jaspers et al. (2019) and Løseth et al. (2019), and cut into small pieces using PFAS-free stainless-steel scissors. For the serum, 10 µL was used for extraction, and for the soil and nettle samples, 300 ± 100 mg (DW for soil and WW for nettles) of material was used. Invertebrates and nettles were homogenised prior to extraction using a TissueLyser LT with stainless beads of 5 mm (50^{-s}) for one minute, and samples were then weighed into 50 mL PP tubes.

PFAS analysis: chemical extraction

Each sample was spiked with 10 ng of a mass-labelled internal standard mixture (MPFAC-MXA, Wellington Laboratories, Guelph, ON, Canada) of seven mass-labelled (^{13}C) perfluoroalkylcarboxylic acids (C_4, C_6, C_8, C_9, C_{10}, C_{11}, and C_{12}) and two mass-labelled (^{18}O and ^{13}C) perfluoroalkylsulfonates (C_6 and C_8). Hereafter, 10 mL of acetonitrile (ACN; HPLC gradient grade, Acros Organics BVBA, Geel, Belgium) was added to all samples, except for the feathers, to which 10 mL of methanol (MeOH; HPLC gradient grade, VWR International, Belgium) was added. After this, all samples were vortex-mixed and sonicated for 3 x 10 min, with vortex-mixing in between. All samples, except the feathers, were then extracted overnight on a shaking plate (135 rpm) at room temperature. The feather samples were left overnight in a dark environment at room temperature. Afterwards, samples were centrifuged (4°C, 10 min, 2400 rpm, Eppendorf centrifuge 5804R) and the supernatant was transferred into 14 mL PP tubes. From here, all samples followed different procedures. The protocol for the feathers was based on the protocol of Groffen et al. (2021), where samples were dried completely using a rotational vacuum concentrator (Eppendorf Concentrator 5301) and then reconstituted with 200 µL of 2% ammonium hydroxide (Thermo Scientific, Belgium; diluted in ACN). Plant and invertebrate samples were extracted based on the protocol of Powley et al. (2005), where samples were dried until 0.5 mL using a rotational vacuum concentrator (Eppendorf Concentrator 5301) and transferred to tubes containing approximately 25 mg of Envi-Carb graphitised carbon adsorbent (Supelclean ENVI-Carb, Sigma-Aldrich, Overijse, Belgium), to which 50 µL of glacial acetic acid (Fisher Scientific, Merelbeke, Belgium) was added. The 14 mL tubes were rinsed twice using 250 µL of ACN, which was added to the same Eppendorf tube. Samples were then vortex-mixed for 1 min and centrifuged for 10 min (10,000 rpm, 4°C, Eppendorf Centrifuge 5415 R). Supernatant was then transferred into a new microcentrifuge tube and dried completely in the rotational vacuum concentrator. The dried eluent was reconstituted with 200 µL of 2% ammonium hydroxide diluted in ACN and vortex-mixed. Finally, soil and serum samples were loaded into Chromabond HR-XAW Solid Phase Extraction (SPE) cartridges (Machery-Nagel, Düren, Germany) that were preconditioned with 5 mL of ACN and 5 mL of Milli-Q water (Groffen et al., 2019b). After loading the samples, the cartridges were washed with 5 mL of a 25 mM ammonium acetate solution and 2 mL of ACN. The cartridges were then eluted with 2 x 1 mL of a 2% ammonium hydroxide solution. The eluent was dried using a rotational vacuum concentrator (Eppendorf Concentrator 5301). After these different procedures, all dried eluents were reconstituted with 200 µL of 2% ammonium hydroxide solution diluted in ACN and filtered using an Ion Chromatography Acrodisc 13 mm Syringe Filter with a 0.2 µm supor polyethersulfone (PES) membrane (VWR International, Belgium) attached to a PP auto-injector vial.

PFAS analysis: UPLC-MS/MS analysis and quantification

All samples were analysed for 29 PFASs using ultra-performance liquid chromatography-coupled tandem electrospray (negative) mass spectrometry (UPLC-ESI-MS/MS). The following PFASs were selected as target analytes: eleven PFCAs (PFBA, PFPeA, PFHxA, PFHpA, PFOA, PFNA, PFDA, PFUnDA, PFDoDA, PFTrDA, and PFTeDA), six PFSAs (PFBS, PFPeS, PFHxS, PFHpS, PFOS, and PFDS), three fluorotelomer sulfonates (4:2, 6:2, and 8:2 FTS), sodiumdodecafluoro-3H-4,8-dioxanonanoate (NaDONA), the major and minor components of F-53B (9Cl-PF3ONS and 11Cl-PF3OUdS), GenX (HFPO-DA), three perfluoroether/polyether-carboxylic acids (PF4OPeA, PF5OHxA, and 3,6-OPFHpA), a perfluoroethersulfonate (PFEESA), and perfluorobutanesulfonamide (FBSA; full names in Table S2). An ACQUITY BEH C18 column (2.1 x 50 mm; 1.7 µm) was used to separate the analytes. To retain any PFAS contamination originating from the system, an ACQUITY BEH C18 pre-column (2.1 x 30 mm; 1.7 µm) was placed between the solvent mixer and the injector. The mobile phase solvents used were 0.1% formic acid in water solution (HPLC grade, VWR International, Belgium) and 0.1% formic acid (LC/MS grade Fisher Chemical, Merelbeke, Belgium) in ACN solution, using a flow rate of 450 µL/min and an injection volume of 6 µL (partial loop). The gradient started at 65% A, decreased to 0% A in 3.4 min, and returned to 65% A at 4.7 min. PFASs were identified and quantified using multiple reaction monitoring (MRM) of 2 diagnostic transitions per target analyte, as validated by Groffen et al. (2021).

Table S1: Total organic carbon (TOC) content per soil sample taken at the corresponding nest box numbers. TOC values are given in percentages.

Nestbox number	TOC content soil (%)
**Vlietbos**	
VB38	1.18
VB32	2.05
VB17A1	2.77
VB15B	2.22
VB35B	5.50
VB15	1.50
VB16B	2.03
VB46	1.88
VB35	2.12
VB29	5.87
VB55	5.17
VB17A2	2.47
VB20	2.21
VB17B	1.75
VB19	2.24
VB70A	2.77
VB19B	1.87
VB49A	4.62
VB17	2.44
**Blokkersdijk/3M**	
BD1	1.45
BD2	2.31
BD3	1.65
BD4	1.15
BD5	4.73

BD6	3.78

Table S1 continued: Total organic carbon (TOC) content per soil sample taken at the corresponding nest box numbers. TOC values are given in percentages.

Nestbox number	TOC content soil (%)
**Blokkersdijk/3M**	
BD7	2.89
BD8	1.94
BD9	2.84
BD10	2.47
BD11	4.09
BD12	1.87
BD13	1.92
BD14	1.35
BD15	5.82
3M65	1.37
3M66B	0.878
3M9	1.80
3M54	0.858
3M19	0.678
3M1	2.09
3M107	1.86
3M7	1.72
3M30	2.17
3M14	2.19
3M56	0.792
3M65	1.80
3M62	2.07
3M3	2.23
3M16	1.59
3M12	2.38
3M55	1.50
3M108	1.32
3M50	0.989

Table S2: Number of analysed samples at both sampling locations

	Blokkersdijk/3M	Vlietbos
**Soil**	114	57
**Nettles**	35	18
**Isopods**	205	75
**Snails**	45	33
**Slugs**	74	72
**Earth worms**	170	85
**Spiders**	43	31
**Great tits**	26	16

Table S3: mean concentrations of the blank samples per batch. The values that are shown are averages of 5 procedural blanks per batch.

	Soil (ng/g DW)	Nettles (ng/g WW)	Invertebrates (ng/g WW)	Plasma (pg/µL)	Feathers (ng/g WW)
**PFBA**	-	-	-	-	-
**PFPeA**	-	-	-	-	-
**PFHxA**	<LOQ – 0.017	-	<LOQ – 0.010	0.012	-
**PFHpA**	-	-	-	-	-
**PFOA**	0.012 – 0.019	0.036	0.014 – 0.082	0.013	-
**PFNA**	-	-	-	-	-
**PFDA**	0.019 – 0.033	0.033	0.012 – 0.033	0.015	<LOQ – 0.019
**PFUnDA**	-	-	0.007 – 0.014	-	-
**PFDoDA**	-	0.097	0.012 – 0.097	0.019	-
**PFTrDA**	-	-	0.014 – 0.032	-	-
**PFTeDA**	-	-	<LOQ – 0.014	-	-
**PFBS**	-	-	-	-	-
**PFPeS**	-	-	-	-	-
**PFHxS**	-	-	-	-	-
**PFHpS**	-	-	-	-	-
**PFOS**	<LOQ – 0.022	-	0.054 – 0.200	-	-
**PFDS**	-	-	-	-	-
**FBSA**	-	-	-	-	-
**4:2 FTS**	-	-	-	-	-
**6:2 FTS**	0.190 – 0.538	0.179	<LOQ – 0.179	-	-
**8:2 FTS**	-	-	-	-	-
**NaDONA**	-	-	-	-	-
**9Cl-PF3ONS**	-	-	-	-	-
**11Cl-PF3OUdS**	-	-	-	-	-
**HFPO-DA**	-	-	-	-	-
**PF4OPeA**	-	-	-	-	-
**PF5OHxA**	-	-	-	-	-
**3,6-OPFHpA**	-	-	-	-	-
**PFEESA**	-	-	-	-	-

Table S4: Limit of quantification (LOQ) of all PFAS analysed in the different matrices evaluated: soil (ng/g DW), nettles (ng/g WW), invertebrates (ng/g WW), plasma (pg/µL) and feathers (ng/g WW).

Compound	Full name	Soil LOQ	Nettles LOQ	Invertebrates LOQ	Plasma LOQ	Feathers LOQ
PFBA	Perfluorobutanoic acid	0.661	0.626	0.944	7.54	2.79
PFPeA	Perfluoropentanoic acid	0.315	0.238	0.814	2.59	0.53
PFHxA	Perfluorohexanoic acid	0.571	0.678	1.47	11.6	4.53
PFHpA	Perfluoroheptanoic acid	0.567	0.076	0.459	4.92	5.55
PFOA	Perfluorooctanoic acid	0.352	0.403	0.611	8.84	0.86
PFNA	Perfluorononanoic acid	0.120	0.126	0.297	1.51	1.42
PFDA	Perfluorododecanoic acid	0.279	0.273	0.666	6.29	1.49
PFUnDA	Perfluoroundecanoic acid	0.264	0.209	0.947	1.08	1.98
PFDoDA	Perfluorododecanoic acid	0.548	0.538	1.28	10.2	2.20
PFTrDA	Perfluorotridecanoic acid	0.235	0.224	0.616	7.86	1.64
PFTeDA	Perfluorotetradecanoic acid	0.947	0.630	0.795	5.67	1.61
PFBS	Perfluorobutanesulfonic acid	0.734	1.25	4.05	387	7.48
PFPeS	Perfluoropentanesulfonic acid	0.224	0.264	0.817	24.1	2.42
PFHxS	Perfluorohexanesulfonic acid	1.65	2.41	4.09	228	3.93
PFHpS	Perfluoroheptanesulfonic acid	0.727	0.693	4.25	33.4	8.32
PFOS	Perfluorooctanesulfonic acid	0.549	0.404	0.465	11.8	1.48
PFDS	Perfluorododecanesulfonic acid	1.73	0.784	6.26	57.2	8.34
FBSA	Perfluorobutanesulfonamide	0.367	0.549	0.938	23.6	3.07
4:2 FTS	4:2 fluorotelomer sulfonic acid	0.285	1.60	0.867	201	10
6:2 FTS	6:2 fluorotelomer sulfonic acid	0.638	0.824	0.400	155	6.27
8:2 FTS	8:2 fluorotelomer sulfonic acid	1.44	0.527	0.520	123	5.37
NaDONA	3H-perfluoro-4,8-dioxanonanoic acid	0.471	0.086	0.118	2.93	0.29
9Cl-PF3ONS	Perfluoro(2-((6-chlorohexyl)oxy)ethanesulfonic acid	0.312	0.264	0.564	13.4	1.67
11Cl-PF3OUdS	11-chloroperfluoro-3-oxadecanesulfonic acid	0.685	0.373	0.607	10.9	1.08
HFPO-DA	Perfluoro-2-methyl-3-oxahexanoic acid	1.84	1.42	0.948	237	18
PF4OPeA	Perfluoro-4-oxapentanoic acid	0.134	0.743	0.435	13.9	1.73
PF5OHxA	Perfluoro-4-methoxybutanoic acid	0.296	1.39	0.856	15.2	1.99
3,6-OPFHpA	Perfluoro-3.6-dioxaheptanoic acid	0.333	1.58	1.23	15.8	2.06
PFEESA	Perfluoro(2-ethoxyethane) sulfonic acid	0.857	0.118	0.189	16.3	2.03

Table S5: percentages of the recoveries of the individual mass-labelled internal standards per matrix analysed in the study.

% Recovery	mPFBA	mPFHxA	mPFHxS	mPFOA	mPFNA	mPFDA	mPFUnDA	mPFDoDA	mPFOS
**Soil**	51.1	43.5	80.1	53.3	70.5	56.0	50.4	67.3	64.9
**Nettles**	35.0	38.3	59.7	42.5	58.8	53.8	46.1	53.3	52.9
**Invertebrates**	38.1	35.0	77.4	49.1	59.4	50.4	52.4	76.9	67.7
**Plasma**	57.2	33.3	23.7	39.8	41.0	43.1	31.1	37.1	29.7
**Feathers**	52.8	38.8	88.6	52.1	75.2	54.6	74.2	94.4	94.7

Table S6: median and mean PFAS concentrations (ng/g DW), ranges and detection frequencies of the topsoil layer (0-10 cm), N = 18 Vlietbos, N = 33 Blokkersdijk/3M

Top soil layer 0-10 cm		PFBA	PFPeA	PFHxA	PFHpA	PFOA	PFNA	PFDA	PFUnDA	PFDoDA	PFTrDA	PFTeDA	PFBS	PFHxS	PFHpS	PFOS	PFDS	FBSA	6:2 FTS
LOQ		0.661	0.315	0.571	0.567	0.352	0.120	0.279	0.264	0.548	0.235	0.947	0.734	1.65	0.727	0.549	1.73	0.367	0.638
Vlietbos	Median	<LOQ	<LOQ	0.638	<LOQ	0.996	0.199	0.643	<LOQ	0.676	<LOQ	<LOQ	<LOQ	<LOQ	<LOQ	9.36	<LOQ	<LOQ	1.06
	Mean	<LOQ	<LOQ	<LOQ	<LOQ	1.10	0.220	0.601	<LOQ	0.570	<LOQ	<LOQ	<LOQ	<LOQ	<LOQ	10.6	<LOQ	<LOQ	2.69
	Range	<LOQ – 2.00	<LOQ – 0.342	<LOQ – 1.28	<LOQ	<LOQ – 4.53	<LOQ – 0.711	<LOQ – 0.937	<LOQ – 0.488	<LOQ – 1.15	<LOQ – 0.652	<LOQ	<LOQ	<LOQ	<LOQ	<LOQ – 38.7	<LOQ	<LOQ	<LOQ – 12.9
	Freq	28	5	56	0	89	83	89	50	72	39	0	0	0	0	89	0	0	56
Blokkersdijk/3M	Median	2.26	0.462	1.56	<LOQ	6.40	0.608	1.27	0.563	2.37	1.32	<LOQ	<LOQ	<LOQ	<LOQ	279	<LOQ	<LOQ	1.06
	Mean	5.33	3.22	9.03	17.8	123	1.39	18.8	2.25	10.5	2.86	2.34	1.79	2.39	1.43	840	16.5	1.15	4.61
	Range	<LOQ – 31.9	<LOQ – 23.4	<LOQ – 60.4	<LOQ – 151	<LOQ – 880	<LOQ – 5.89	<LOQ – 482	<LOQ – 13.2	<LOQ – 86.4	<LOQ – 14.3	<LOQ – 13.9	<LOQ – 13.4	<LOQ – 40.3	<LOQ – 10.0	39.7 – 3599	<LOQ – 138	<LOQ – 8.44	<LOQ – 25.0
	Freq	82	61	85	48	97	91	97	82	89	82	52	30	9	36	100	39	48	52

Table S7: median and mean PFAS concentrations (ng/g DW), ranges and detection frequencies of soil layer 3 (20-30cm), N = 18 Vlietbos, N = 35 Blokkersdijk/3M

Soil layer approx. 20-30 cm deep		PFBA	PFPeA	PFHxA	PFHpA	PFOA	PFNA	PFDA	PFUnDA	PFDoDA	PFTrDA	PFTeDA	PFBS	PFPeS	PFHxS	PFHpS	PFOS	PFDS	FBSA	6:2 FTS
LOQ		0.661	0.315	0.571	0.567	0.352	0.120	0.279	0.264	0.548	0.235	0.947	0.734	0.224	1.65	0.727	0.549	1.73	0.367	0.638
Vlietbos	Median	<LOQ	<LOQ	<LOQ	<LOQ	0.937	0.170	<LOQ	<LOQ	<LOQ	<LOQ	<LOQ	<LOQ	<LOQ	<LOQ	<LOQ	11.6	<LOQ	0.798	<LOQ
	Mean	<LOQ	<LOQ	<LOQ	<LOQ	1.38	0.168	<LOQ	<LOQ	<LOQ	<LOQ	<LOQ	1.21	<LOQ	<LOQ	<LOQ	13.8	<LOQ	1.12	<LOQ
	Range	<LOQ – 2.99	<LOQ	<LOQ	<LOQ	0.375 – 3.89	<LOQ – 0.545	<LOQ	<LOQ	<LOQ – 0.627	<LOQ	<LOQ	<LOQ – 11.4	<LOQ	<LOQ	<LOQ – 0.923	3.98 – 37.4	<LOQ	<LOQ – 3.37	<LOQ
	Freq	26	0	0	0	100	63	0	0	5	0	0	37	0	0	5	100	0	89	0
Blokkersdijk/3M	Median	2.51	<LOQ	<LOQ	<LOQ	8.44	0.557	<LOQ	<LOQ	0.896	<LOQ	<LOQ	1.71	<LOQ	1.90	2.74	248	2.09	4.39	<LOQ
	Mean	4.64	1.19	9.59	12.3	85.1	1.15	3.47	1.25	6.65	1.74	1.58	3.86	0.347	4.45	8.18	520	16.4	5.40	1.80
	Range	<LOQ – 32.9	<LOQ – 11.8	<LOQ – 75.8	<LOQ – 69.5	0.963 – 494	<LOQ – 6.10	<LOQ – 84.6	<LOQ – 6.98	<LOQ – 38.3	<LOQ – 10.7	<LOQ – 9.71	<LOQ – 29.5	<LOQ – 1.85	<LOQ – 25.1	<LOQ – 60.0	17.4 – 2800	<LOQ – 95.8	0.404 – 32.1	<LOQ – 28.8
	Freq	86	37	45	43	100	80	37	29	60	31	26	80	37	54	77	100	51	100	20

Table S8: median and mean PFAS concentrations (ng/g DW), ranges and detection frequencies of soil layer 5 (40-50 cm), N = 17 Vlietbos, N = 35 Blokkersdijk/3M

Soil layer approx. 40-50 cm deep		PFBA	PFPeA	PFHxA	PFHpA	PFOA	PFNA	PFDA	PFUnDA	PFDoDA	PFTrDA	PFTeDA	PFBS	PFPeS	PFHxS	PFHpS	PFOS	PFDS	FBSA	6:2 FTS
LOQ		0.661	0.315	0.571	0.567	0.352	0.120	0.279	0.264	0.548	0.235	0.947	0.734	0.224	1.65	0.727	0.549	1.73	0.367	0.638
Vlietbos	Median	<LOQ	<LOQ	<LOQ	<LOQ	1.05	<LOQ	0.518	<LOQ	0.649	<LOQ	<LOQ	<LOQ	<LOQ	<LOQ	<LOQ	5.29	<LOQ	<LOQ	1.02
	Mean	<LOQ	<LOQ	<LOQ	<LOQ	1.25	0.128	0.542	<LOQ	0.602	<LOQ	<LOQ	<LOQ	<LOQ	<LOQ	<LOQ	8.31	<LOQ	<LOQ	1.38
	Range	<LOQ – 0.693	<LOQ	<LOQ – 0.798	<LOQ	<LOQ – 3.35	<LOQ – 0.445	<LOQ – 1.03	<LOQ – 0.489	<LOQ – 1.52	<LOQ – 0.490	<LOQ	<LOQ	<LOQ	<LOQ	<LOQ	<LOQ – 32.1	<LOQ	<LOQ	<LOQ – 3.18
	Freq	6	0	35	0	94	41	82	29	71	12	0	0	0	0	0	94	0	0	53
Blokkersdijk/3M	Median	1.52	<LOQ	1.10	<LOQ	7.98	0.602	1.07	0.496	1.22	0.250	<LOQ	<LOQ	<LOQ	<LOQ	<LOQ	223	<LOQ	<LOQ	<LOQ
	Mean	11.6	6.30	20.7	26.9	157	1.13	6.87	1.96	8.61	2.16	1.49	2.36	0.386	9.75	88.8	663	11.7	6.77	1.19
	Range	<LOQ – 307	<LOQ – 163	<LOQ – 510	<LOQ – 565	<LOQ – 3202	<LOQ – 4.65	<LOQ – 131	<LOQ – 22.1	<LOQ – 53.0	<LOQ – 10.9	<LOQ – 10.1	<LOQ – 19.5	<LOQ – 11.9	<LOQ – 204	<LOQ – 3033	<LOQ – 3744	<LOQ – 67.3	<LOQ – 214	<LOQ – 10.2
	Freq	37	46	18	46	100	77	91	80	83	51	34	26	11	20	37	97	40	29	37

Table S9: median and mean PFAS concentrations (ng/g DW), ranges and detection frequencies of soil layer 7 (60-70 cm), N = 18 Vlietbos, N = 33 Blokkersdijk/3M

Soil layer approx. 60-70 cm deep		PFBA	PFPeA	PFHxA	PFHpA	PFOA	PFNA	PFDA	PFUnDA	PFDoDA	PFTrDA	PFTeDA	PFBS	PFPeS	PFHxS	PFHpS	PFOS	PFDS	FBSA	6:2 FTS
LOQ		0.661	0.315	0.571	0.567	0.352	0.120	0.279	0.264	0.548	0.235	0.947	0.734	0.224	1.65	0.727	0.549	1.73	0.367	0.638
Vlietbos	Median	<LOQ	<LOQ	<LOQ	<LOQ	1.22	<LOQ	<LOQ	<LOQ	<LOQ	<LOQ	<LOQ	0.898	<LOQ	<LOQ	<LOQ	9.27	<LOQ	0.920	<LOQ
	Mean	<LOQ	<LOQ	<LOQ	<LOQ	1.79	0.127	<LOQ	<LOQ	<LOQ	<LOQ	<LOQ	0.906	<LOQ	<LOQ	<LOQ	10.5	<LOQ	1.55	<LOQ
	Range	<LOQ – 1.84	<LOQ	<LOQ	<LOQ	0.718 – 5.55	<LOQ – 0.395	<LOQ	<LOQ	<LOQ – 1.94	<LOQ	<LOQ	<LOQ – 4.61	<LOQ	<LOQ	<LOQ – 1.84	3.15 – 24.9	<LOQ	<LOQ – 7.03	<LOQ
	Freq	33	0	0	0	100	39	0	0	6	0	0	61	0	0	33	100	0	94	0
Blokkersdijk/3M	Median	2.53	<LOQ	<LOQ	<LOQ	8.57	0.374	<LOQ	<LOQ	<LOQ	<LOQ	<LOQ	2.34	<LOQ	2.86	5.17	162	<LOQ	2.70	<LOQ
	Mean	3.64	0.664	6.69	8.46	58.5	0.856	2.96	1.04	4.45	1.26	1.27	4.07	0.330	4.44	9.94	507	14.4	3.91	1.95
	Range	<LOQ – 14.0	<LOQ – 4.45	<LOQ – 53.4	<LOQ – 45.1	0.888 – 340	<LOQ – 3.45	<LOQ – 66.2	<LOQ – 4.94	<LOQ – 36.8	<LOQ – 10.7	<LOQ – 8.49	<LOQ – 18.5	<LOQ – 2.45	<LOQ – 34.6	<LOQ – 58.7	10.9 – 2635	<LOQ – 135	<LOQ – 17.9	<LOQ – 17.3
	Freq	88	39	48	36	100	79	45	33	36	36	30	76	33	70	91	100	33	97	18

Table S10: median and mean PFAS concentrations (ng/g DW), ranges and detection frequencies of soil layer 10 (90-100 cm), N = 18 Vlietbos, N = 29 Blokkersdijk/3M

Soil layer approx. 90-100 cm deep		PFBA	PFPeA	PFHxA	PFHpA	PFOA	PFNA	PFDA	PFUnDA	PFDoDA	PFTrDA	PFTeDA	PFBS	PFPeS	PFHxS	PFHpS	PFOS	PFDS	FBSA	6:2 FTS
LOQ		0.661	0.315	0.571	0.567	0.352	0.120	0.279	0.264	0.548	0.235	0.947	0.734	0.224	1.65	0.727	0.549	1.73	0.367	0.638
Vlietbos	Median	<LOQ	<LOQ	<LOQ	<LOQ	1.08	<LOQ	0.553	<LOQ	<LOQ	<LOQ	<LOQ	<LOQ	<LOQ	<LOQ	<LOQ	2.48	<LOQ	<LOQ	0.799
	Mean	<LOQ	<LOQ	<LOQ	<LOQ	1.58	<LOQ	0.571	<LOQ	<LOQ	<LOQ	<LOQ	<LOQ	<LOQ	<LOQ	<LOQ	2.91	<LOQ	<LOQ	1.49
	Range	<LOQ – 1.67	<LOQ	<LOQ – 0.840	<LOQ	0.490 – 7.23	<LOQ – 0.250	<LOQ – 0.839	<LOQ – 0.336	<LOQ – 1.67	<LOQ – 0.366	<LOQ	<LOQ – 9.70	<LOQ	<LOQ	<LOQ	0.950 – 8.09	<LOQ	<LOQ	<LOQ – 7.65
	Freq	5	0	22	0	100	17	100	22	39	11	0	5	0	0	0	100	0	0	56
Blokkersdijk/3M	Median	1.84	0.401	1.01	<LOQ	4.1	0.560	1.34	0.326	0.724	<LOQ	<LOQ	<LOQ	<LOQ	<LOQ	<LOQ	257	<LOQ	<LOQ	<LOQ
	Mean	2.37	1.81	7.06	13.5	84.1	3.47	17.5	1.48	7.48	<LOQ	1.29	3.12	<LOQ	3.33	6.30	2679	28.6	0.468	4.56
	Range	<LOQ – 14.7	<LOQ – 22.3	<LOQ – 87.7	<LOQ – 135	<LOQ – 937	<LOQ – 71.1	<LOQ – 356	<LOQ – 10.4	<LOQ – 34.5	<LOQ – 0.813	<LOQ – 7.49	<LOQ – 21.3	<LOQ – 3.89	<LOQ – 87.9	<LOQ – 125	11.5 – 58213	<LOQ – 604	<LOQ – 6.47	<LOQ – 58.0
	Freq	62	55	69	41	90	69	83	52	62	21	31	69	3	7	41	100	34	14	34

Table S11: median and mean PFAS concentrations (ng/g WW), ranges and detection frequencies in the nettles sampled at Vlietbos (N = 18) and Blokkersdijk/3M (N = 35)

Nettles		PFBA	PFPeA	PFHxA	PFHpA	PFOA	PFNA	PFDA	PFUnDA	PFDoDA	PFTrDA	PFTeDA	PFBS	PFPeS	PFHxS	PFHpS	PFOS	PFDS	FBSA	4:2 FTS	6:2 FTS	NaDONA
LOQ		0.626	0.238	0.678	0.076	0.403	0.126	0.273	0.209	0.538	0.224	0.630	1.25	0.264	2.41	0.693	0.404	0.784	0.549	1.60	0.824	0.086
Vlietbos	Median	<LOQ	<LOQ	<LOQ	<LOQ	0.512	<LOQ	0.436	<LOQ	<LOQ	<LOQ	<LOQ	<LOQ	<LOQ	<LOQ	<LOQ	0.805	<LOQ	<LOQ	<LOQ	<LOQ	<LOQ
	Mean	<LOQ	<LOQ	<LOQ	<LOQ	0.537	<LOQ	0.396	<LOQ	<LOQ	<LOQ	<LOQ	<LOQ	<LOQ	<LOQ	<LOQ	0.956	<LOQ	<LOQ	<LOQ	3.34	<LOQ
	Range	<LOQ – 1.38	<LOQ	<LOQ	<LOQ	<LOQ – 1.03	<LOQ – 0.198	<LOQ – 0.661	<LOQ – 0.398	<LOQ – 1.26	<LOQ – 0.363	<LOQ	<LOQ	<LOQ	<LOQ	<LOQ	<LOQ – 5.04	<LOQ	<LOQ	<LOQ	<LOQ – 26.7	<LOQ – 0.240
	Freq (%)	5	0	0	0	67	5	78	5	22	5	0	0	0	0	0	78	0	0	0	44	44
Blokkersdijk/3M	Median	13.0	0.367	<LOQ	<LOQ	2.66	<LOQ	0.619	<LOQ	1.50	<LOQ	<LOQ	<LOQ	<LOQ	<LOQ	<LOQ	31.0	<LOQ	<LOQ	<LOQ	<LOQ	<LOQ
	Mean	20.6	1.98	2.87	0.631	33.7	0.331	0.641	0.649	11.5	2.96	1.62	3.83	<LOQ	10.6	1.85	161	9.96	2.64	<LOQ	2.10	<LOQ
	Range	<LOQ – 88.8	<LOQ – 11.69	<LOQ – 19.6	<LOQ – 3.51	0.488 – 172	<LOQ – 1.95	<LOQ – 1.71	<LOQ – 9.72	<LOQ – 185	<LOQ – 44.9	<LOQ – 19.8	<LOQ – 27.5	<LOQ – 1.28	<LOQ – 88.1	<LOQ – 15.6	<LOQ - 616	<LOQ – 199	<LOQ – 16.6	<LOQ – 3.41	<LOQ – 32.4	<LOQ – 0.177
	Freq (%)	91	65	49	37	100	37	94	49	71	46	31	43	26	29	34	97	40	49	17	17	11

Table S12: median and mean PFAS concentrations (ng/g DW), ranges and detection frequencies in the isopods sampled at Vlietbos (N = 16) and Blokkersdijk/3M (N = 36), each value was a mean value of 5 different isopods per nest box

Isopods		PFBA	PFPeA	PFHxA	PFHpA	PFOA	PFNA	PFDA	PFUnDA	PFDoDA	PFTrDA	PFTeDA	PFBS	PFPeS	PFHxS	PFHpS	PFOS	PFDS	FBSA	6:2 FTS	8:2 FTS
LOQ		0.949	0.814	1.47	0.459	0.611	0.297	0.666	0.947	1.28	0.616	0.795	4.05	0.817	4.09	4.25	0.465	6.26	0.938	0.400	0.520
Vlietbos	Median	<LOQ	<LOQ	3.71	<LOQ	4.33	<LOQ	5.30	2.58	4.49	0.800	<LOQ	<LOQ	<LOQ	<LOQ	<LOQ	1.11	<LOQ	<LOQ	<LOQ	<LOQ
	Mean	<LOQ	<LOQ	3.67	<LOQ	5.36	<LOQ	5.76	2.37	4.96	0.708	<LOQ	<LOQ	<LOQ	<LOQ	<LOQ	2.11	<LOQ	<LOQ	<LOQ	<LOQ
	Range	<LOQ – 5.71	<LOQ	<LOQ – 6.79	<LOQ	1.06 – 14.4	<LOQ	3.43 – 11.6	0.984 – 4.54	3.00 – 7.60	<LOQ – 1.83	<LOQ – 2.10	<LOQ – 10.1	<LOQ	<LOQ	<LOQ	<LOQ – 15.4	<LOQ	<LOQ	<LOQ	<LOQ
	Freq	13	0	88	0	100	0	100	100	100	63	31	6	0	0	0	94	0	0	0	0
Blokkersdijk/3M	Median	12.1	1.38	7.76	<LOQ	12.0	<LOQ	6.50	8.41	13.1	6.99	2.02	5.19	<LOQ	<LOQ	<LOQ	65.5	<LOQ	<LOQ	<LOQ	<LOQ
	Mean	24.8	13.4	25.8	1.87	34.7	<LOQ	9.53	9.35	229	86.2	49.0	18.0	<LOQ	4.62	<LOQ	313	46.0	3.24	42.0	<LOQ
	Range	<LOQ – 140	<LOQ – 75.0	<LOQ – 157	<LOQ – 18.1	1.70 – 258	<LOQ – 1.44	1.08 – 57.8	1.37 – 33.8	3.71 – 2262	0.909 – 446	<LOQ – 217	<LOQ – 96.0	<LOQ – 1.05	<LOQ – 132	<LOQ – 6.00	4.83 – 2677	<LOQ – 723	<LOQ – 17.4	<LOQ – 1074	<LOQ – 7.51
	Freq	72	78	83	17	100	19	100	100	100	100	51	56	3	6	8	100	36	44	14	3

Table S13: median and mean PFAS concentrations (ng/g WW), ranges and detection frequencies in the earthworms sampled at Vlietbos (N = 17) and Blokkersdijk/3M (N = 28), each value was a mean value of 5 different earthworms per nest box

Earthworms		PFBA	PFPeA	PFHxA	PFHpA	PFOA	PFNA	PFDA	PFUnDA	PFDoDA	PFTrDA	PFTeDA	PFBS	PFPeS	PFHxS	PFHpS	PFOS	PFDS	FBSA	6:2 FTS	8:2 FTS
LOQ		0.949	0.814	1.47	0.459	0.611	0.297	0.666	0.947	1.28	0.616	0.795	4.05	0.817	4.09	4.25	0.465	6.26	0.938	0.400	0.520
Vlietbos	Median	1.59	<LOQ	<LOQ	<LOQ	1.04	<LOQ	1.07	<LOQ	3.45	2.96	3.82	5.82	<LOQ	<LOQ	<LOQ	21.3	<LOQ	1.93	<LOQ	<LOQ
	Mean	1.44	<LOQ	<LOQ	<LOQ	5.81	0.250	1.46	<LOQ	3.49	4.02	6.05	7.44	<LOQ	<LOQ	<LOQ	37.8	<LOQ	1.93	3.48	<LOQ
	Range	<LOQ – 6.10	<LOQ – 1.92	<LOQ – 3.22	<LOQ – 1.38	<LOQ – 83.7	<LOQ – 1.64	<LOQ – 3.58	<LOQ – 4.03	<LOQ – 7.12	0.904 – 9.02	<LOQ – 25.0	<LOQ – 25.0	<LOQ	<LOQ	<LOQ	5.93 – 192	<LOQ	<LOQ – 6.68	<LOQ – 55.5	<LOQ
	Freq	53	12	41	12	94	18	94	53	94	100	88	71	0	0	0	100	0	59	12	0
Blokkersdijk/3M	Median	14.0	1.08	2.97	<LOQ	7.27	0.613	2.46	2.71	46.9	44.1	38.7	81.2	<LOQ	20.2	33.5	3900	134	33.2	<LOQ	<LOQ
	Mean	17.8	5.74	10.9	4.80	39.5	1.76	5.86	22.8	327	219	179	150	8.23	194	72.0	6098	1344	46.0	5.97	0.360
	Range	<LOQ – 65.3	<LOQ – 62.0	<LOQ – 87.5	<LOQ – 59.4	1.00 – 284	<LOQ – 11.3	<LOQ – 30.9	<LOQ – 280	6.54 – 2729	5.13 – 1160	4.83 – 1189	<LOQ – 1364	<LOQ – 58.4	<LOQ – 2301	<LOQ – 377	169 – 30460	7.97 – 14967	6.83 – 206	<LOQ – 66.9	<LOQ – 4.03
	Freq	89	79	79	21	100	82	96	89	100	100	100	96	36	61	86	100	100	100	32	11

Table S14: median and mean PFAS concentrations (ng/g WW), ranges and detection frequencies in the snails sampled at Vlietbos (N = 8) and Blokkersdijk/3M (N = 15), each value was a mean value of 5 different snails per nest box

snails		PFBA	PFPeA	PFHxA	PFHpA	PFOA	PFNA	PFDA	PFUnDA	PFDoDA	PFTrDA	PFTeDA	PFBS	PFPeS	PFHxS	PFHpS	PFOS	PFDS	FBSA	4:2 FTS	6:2 FTS	8:2 FTS
LOQ		0.949	0.814	1.47	0.459	0.611	0.297	0.666	0.947	1.28	0.616	0.795	4.05	0.817	4.09	4.25	0.465	6.26	0.938	0.867	0.400	0.520
Vlietbos	Median	<LOQ	<LOQ	<LOQ	<LOQ	1.41	0.309	1.66	<LOQ	2.48	1.41	0.839	<LOQ	<LOQ	<LOQ	<LOQ	19.9	<LOQ	1.88	<LOQ	0.626	<LOQ
	Mean	<LOQ	<LOQ	<LOQ	<LOQ	1.38	0.422	1.42	<LOQ	2.72	1.91	1.56	<LOQ	<LOQ	<LOQ	<LOQ	28.2	<LOQ	1.77	<LOQ	11.1	1.75
	Range	<LOQ – 1.69	<LOQ	<LOQ – 2.58	<LOQ	<LOQ – 2.79	<LOQ – 1.34	<LOQ – 2.66	<LOQ – 1.29	<LOQ – 9.32	<LOQ – 8.31	<LOQ – 6.91	<LOQ	<LOQ	<LOQ	<LOQ	0.942 – 108	<LOQ	<LOQ – 3.08	<LOQ	<LOQ – 48.0	<LOQ – 11.8
	Freq	38	0	50	0	86	63	75	38	63	63	50	0	0	0	0	100	0	63	0	75	25
Blokkersdijk/3M	Median	39.1	<LOQ	<LOQ	<LOQ	9.41	1.09	3.49	1.64	11.4	4.24	2.62	9.90	<LOQ	<LOQ	4.84	1486	12.2	27.0	<LOQ	<LOQ	<LOQ
	Mean	44.8	2.87	3.57	<LOQ	35.2	4.70	6.12	14.1	66.3	38.3	20.9	15.9	1.77	242	55.2	7687	614	25.9	<LOQ	4.50	2.80
	Range	1.58 – 107	<LOQ – 11.9	<LOQ – 24.1	<LOQ – 1.21	1.56 – 199	<LOQ – 20.0	<LOQ – 26.8	<LOQ – 56.1	<LOQ – 248	<LOQ – 127	<LOQ – 79.3	<LOQ – 88.5	<LOQ – 8.49	<LOQ – 1590	<LOQ – 318	14.1 – 50972	<LOQ – 2893	<LOQ – 57.2	<LOQ – 0.939	<LOQ – 22.4	<LOQ – 19.1
	Freq	100	40	47	27	100	87	93	87	93	93	67	80	33	40	67	100	67	93	13	46	20

Table S15: median and mean PFAS concentrations (ng/g WW), ranges and detection frequencies in the slugs sampled at Vlietbos (N = 14) and Blokkersdijk/3M (N = 18), each value was a mean value of 5 different slugs per nest box

slugs		PFBA	PFPeA	PFHxA	PFHpA	PFOA	PFNA	PFDA	PFUnDA	PFDoDA	PFTrDA	PFTeDA	PFBS	PFPeS	PFHxS	PFHpS	PFOS	PFDS	FBSA	6:2 FTS	8:2 FTS
LOQ		0.949	0.814	1.47	0.459	0.611	0.297	0.666	0.947	1.28	0.616	0.795	4.05	0.817	4.09	4.25	0.465	6.26	0.938	0.400	0.520
Vlietbos	Median	<LOQ	<LOQ	<LOQ	<LOQ	<LOQ	<LOQ	<LOQ	<LOQ	<LOQ	<LOQ	<LOQ	<LOQ	<LOQ	<LOQ	<LOQ	7.90	<LOQ	1.64	<LOQ	<LOQ
	Mean	<LOQ	<LOQ	<LOQ	<LOQ	<LOQ	<LOQ	<LOQ	<LOQ	<LOQ	<LOQ	<LOQ	<LOQ	<LOQ	<LOQ	<LOQ	9.12	<LOQ	2.20	<LOQ	<LOQ
	Range	<LOQ	<LOQ	<LOQ	<LOQ	<LOQ – 1.27	<LOQ	<LOQ – 1.75	<LOQ	<LOQ – 1.88	<LOQ – 1.24	<LOQ	<LOQ	<LOQ	<LOQ	<LOQ	3.33 – 27.3	<LOQ	<LOQ – 7.66	<LOQ – 3.28	<LOQ
	Freq	0	0	0	0	21	0	29	0	29	14	0	0	0	0	0	100	0	86	7	0
Blokkersdijk/3M	Median	3.68	<LOQ	2.08	0.685	13.2	1.87	4.89	3.65	42.5	19.5	10.9	<LOQ	<LOQ	4.50	10.9	1838	105	18.7	<LOQ	<LOQ
	Mean	5.48	0.664	2.38	3.74	34.9	2.85	6.26	15.3	102	26.3	12.9	5.06	<LOQ	19.3	15.5	4197	242	25.4	<LOQ	<LOQ
	Range	1.56 – 22.7	<LOQ – 2.03	<LOQ – 9.87	<LOQ – 32.2	<LOQ – 132	<LOQ – 8.77	1.11 – 18.6	<LOQ – 152	<LOQ – 572	<LOQ – 104	<LOQ – 39.0	<LOQ – 34.9	<LOQ – 1.67	<LOQ – 72.3	<LOQ – 92.8	42.1 – 14780	<LOQ – 1412	3.21 – 78.1	<LOQ – 1.06	<LOQ – 1.15
	Freq	100	39	67	50	94	83	100	67	89	72	72	39	22	50	67	100	72	100	22	17

Table S16: median and mean PFAS concentrations (ng/g WW), ranges and detection frequencies in the spiders sampled at Vlietbos (N = 6) and Blokkersdijk/3M (N = 11), each value was a mean value of 5 different spiders per nest box

spiders		PFBA	PFPeA	PFHxA	PFHpA	PFOA	PFNA	PFDA	PFUnDA	PFDoDA	PFTrDA	PFTeDA	PFBS	PFHxS	PFHpS	PFOS	PFDS	FBSA
LOQ		0.949	0.814	1.47	0.459	0.611	0.297	0.666	0.947	1.28	0.616	0.795	4.05	4.09	4.25	0.465	6.26	0.938
Vlietbos	Median	<LOQ	<LOQ	4.71	<LOQ	6.54	<LOQ	11.2	3.50	7.14	<LOQ	<LOQ	<LOQ	<LOQ	<LOQ	1.73	<LOQ	1.47
	Mean	<LOQ	<LOQ	5.28	<LOQ	6.95	<LOQ	11.3	3.73	8.81	0.489	<LOQ	<LOQ	<LOQ	<LOQ	28.8	<LOQ	1.32
	Range	<LOQ	<LOQ	<LOQ – 12.5	<LOQ	1.92 – 14.0	<LOQ	6.92 – 16.7	1.23 – 7.06	5.10 – 16.4	<LOQ – 1.49	<LOQ	<LOQ – 3.86	<LOQ	<LOQ	<LOQ – 164	<LOQ	<LOQ – 2.68
	Freq	0	0	83	0	100	0	100	100	100	33	0	17	0	0	83	0	50
Blokkersdijk/3M	Median	<LOQ	<LOQ	<LOQ	<LOQ	96.9	<LOQ	26.8	12.2	<LOQ	<LOQ	<LOQ	15.4	<LOQ	<LOQ	679	<LOQ	40.3
	Mean	2.90	<LOQ	12.0	0.777	151	<LOQ	33.4	15.4	72.8	20.5	9.36	46.8	7.98	3.33	1927	83.4	112
	Range	<LOQ – 17.7	<LOQ – 1.04	<LOQ – 104	<LOQ – 4.75	15.1 – 546	<LOQ – 1.26	2.93 – 111	1.35 – 44.8	<LOQ – 399	<LOQ – 109	<LOQ – 48.3	<LOQ – 225	<LOQ – 41.6	<LOQ – 24.7	77.3 – 12379	<LOQ – 633	<LOQ – 732
	Freq	27	9	36	18	100	18	100	100	45	36	45	64	27	27	100	27	82

Table S17: median and mean PFAS concentrations (µg/L), ranges and detection frequencies in the plasma of great tits sampled at Vlietbos (N = 15) and Blokkersdijk/3M (N = 23)

plasma		PFHxA	PFOA	PFNA	PFDA	PFUnDA	PFDoDA	PFTrDA	PFTeDA	PFHpS	PFOS	PFDS	6:2 FTS
LOQ		11.6	8.84	1.51	6.29	1.08	10.2	7.86	5.67	33.4	11.8	57.2	155
Vlietbos	Median	<LOQ	11.0	<LOQ	15.7	<LOQ	<LOQ	<LOQ	<LOQ	<LOQ	<LOQ	<LOQ	<LOQ
	Mean	4.45	11.3	<LOQ	14.2	<LOQ	27.9	<LOQ	<LOQ	<LOQ	1182	<LOQ	170
	Range	<LOQ – 32.2	<LOQ – 47.9	<LOQ	<LOQ – 26.2	<LOQ	<LOQ – 309	<LOQ - 102	<LOQ	<LOQ - 175	<LOQ – 17281	<LOQ - 484	<LOQ – 2556
	Freq	20	53	0	73	0	27	7	0	7	33	7	7
Blokkersdijk/3M	Median	<LOQ	28.3	<LOQ	26.2	<LOQ	87.8	16.6	<LOQ	<LOQ	7554	<LOQ	<LOQ
	Mean	4.14	60.7	6.19	23.4	5.41	201	41.9	7.46	<LOQ	14520	62.1	213
	Range	<LOQ – 33.7	<LOQ – 305	<LOQ – 28.5	<LOQ – 52.0	<LOQ – 25.4	<LOQ – 950	<LOQ – 195	<LOQ – 60.5	<LOQ – 175	196 – 56088	<LOQ – 961	<LOQ – 4896
	Freq	17	87	39	87	30	78	52	13	13	100	13	4

Table S18: median and mean PFAS concentrations (ng/g), ranges and detection frequencies in the feathers sampled at Vlietbos (N = 15) and Blokkersdijk/3M (N = 23)

Feathers		PFBA	PFPeA	PFHxA	PFHpA	PFOA	PFNA	PFDA	PFUnDA	PFDoDA	PFTrDA	PFTeDA	PFBS	PFPeS	PFHxS	PFHpS	PFOS	PFDS	FBSA	6:2 FTS	NaDONA
LOQ		2.79	0.530	4.53	5.55	0.860	1.42	1.49	1.98	2.20	1.64	1.61	7.48	2.42	2.93	8.32	1.48	8.34	3.07	6.27	0.290
Vlietbos	Median	<LOQ	<LOQ	<LOQ	<LOQ	12.2	<LOQ	12.6	5.59	9.36	<LOQ	<LOQ	<LOQ	<LOQ	<LOQ	<LOQ	18.9	<LOQ	<LOQ	<LOQ	<LOQ
	Mean	<LOQ	<LOQ	2.9	<LOQ	11.4	<LOQ	13.2	4.40	10.3	<LOQ	<LOQ	<LOQ	<LOQ	<LOQ	<LOQ	22.6	<LOQ	<LOQ	<LOQ	0.749
	Range	<LOQ	<LOQ	<LOQ – 19.4	<LOQ	1.65 – 33.2	<LOQ	<LOQ – 34.0	<LOQ – 11.4	<LOQ – 32.1	<LOQ	<LOQ	<LOQ	<LOQ	<LOQ	<LOQ	6.73 – 51.4	<LOQ	<LOQ	<LOQ – 42.9	<LOQ – 3.05
	Freq	0	0	20	0	100	0	93	73	87	0	0	0	0	0	0	100	0	0	7	40
Blokkersdijk/3M	Median	36.8	2.00	22.0	<LOQ	41.3	<LOQ	13.8	5.48	11.8	<LOQ	<LOQ	65.7	<LOQ	<LOQ	<LOQ	793	<LOQ	<LOQ	<LOQ	<LOQ
	Mean	67.9	17.4	72.7	33.6	211	1.00	14.2	4.40	26.8	6.20	2.80	75.5	4.20	142	66.4	2828	24.8	<LOQ	3.50	0.740
	Range	<LOQ – 250	<LOQ – 71.8	<LOQ – 346	<LOQ – 138	6.14 – 986	<LOQ – 7.50	<LOQ – 55.6	<LOQ – 10.0	<LOQ – 251	<LOQ – 36.4	<LOQ – 17.9	<LOQ – 296	<LOQ – 52.9	<LOQ – 990	<LOQ – 300	<LOQ – 9590	<LOQ – 127	<LOQ – 18.6	<LOQ – 80.3	<LOQ – 3.80
	Freq	82	52	87	35	100	22	91	61	65	43	17	57	17	26	48	91	30	4	4	35

Table S19: Mean PFAS concentrations found in each matrix at both sampling sites; Blokkersdijk/3M (BD3M) and Vlietbos (VB). Concentrations are expressed in nmol/g, consistent with the units used in the development of the PFAS profiles of each matrix.

Compound	Soil L1		Soil L3		Soil L5		Soil L7		Soil L10		Nettles		Isopods		Earthworms		Snails		Slugs		Spiders		Plasma		Feathers	
	BD3M	VB	BD3M	VB	BD3M	VB	BD3M	VB	BD3M	VB	BD3M	VB	BD3M	VB	BD3M	VB	BD3M	VB	BD3M	VB	BD3M	VB	BD3M	VB	BD3M	VB
PFBA	0.025	0.003	0.022	0.003	0.054	0.002	0.017	0.003	0.012	0.002	0.097	0.002	0.117	0.004	0.083	0.008	0.209	0.004	0.026	/	0.015	/	/	/	0.318	/
PFPeA	0.012	0.0006	0.005	/	0.024	/	0.003	0.0006	0.007	/	0.008	/	0.051	/	0.022	0.002	0.011	/	0.003	/	0.002	/	/	/	0.066	/
PFHxA	0.029	0.002	0.031	/	0.066	0.001	0.022	/	0.023	0.001	0.010	/	0.083	0.012	0.035	0.004	0.012	0.004	0.008	0.002	0.040	0.017	0.028	0.029	0.231	0.015
PFHpA	0.049	/	0.034	/	0.074	/	0.024	/	0.037	/	0.002	/	0.006	/	0.014	0.0009	0.001	/	0.010	/	0.003	/	/	/	0.097	/
PFOA	0.296	0.003	0.206	0.003	0.379	0.003	0.141	0.004	0.203	0.004	0.081	0.001	0.084	0.013	0.095	0.014	0.085	0.003	0.084	0.001	0.364	0.017	0.148	0.032	0.510	0.028
PFNA	0.003	0.0005	0.003	0.0004	0.002	0.0003	0.002	0.0003	0.007	0.0002	0.0007	0.0002	0.0006	/	0.004	0.0007	0.010	0.001	0.006	/	0.0006	/	0.014	/	0.003	/
PFDA	0.037	0.001	0.007	/	0.013	0.001	0.006	/	0.034	0.001	0.001	0.0008	0.019	0.011	0.011	0.003	0.012	0.003	0.012	0.001	0.065	0.022	0.047	0.029	0.028	0.026
PFUnDA	0.004	0.0005	0.002	/	0.004	0.0004	0.002	0.0006	0.003	0.0003	0.001	0.0002	0.017	0.004	0.041	0.002	0.025	0.001	0.027	0.0009	0.027	0.007	0.010	/	0.008	0.009
PFDoDA	0.017	0.001	0.011	0.0005	0.014	0.001	0.007	/	0.012	0.0008	0.019	0.0007	0.373	0.008	0.533	0.006	0.108	0.005	0.166	0.001	0.119	0.014	0.329	0.051	0.044	0.017
PFTrDA	0.004	0.0003	0.003	/	0.003	0.0002	0.002	/	0.0003	0.0002	0.005	0.0002	0.130	0.001	0.329	0.006	0.058	0.003	0.040	0.0006	0.031	0.001	0.066	0.016	0.010	/
PFTeDA	0.004	/	0.003	/	0.002	/	0.002	/	0.002	/	0.003	/	0.069	0.0009	0.250	0.009	0.029	0.002	0.018	0.0006	0.013	/	0.014	/	0.005	/
PFBS	0.007	0.001	0.013	0.005	0.009	/	0.014	0.003	0.011	0.003	0.014	/	0.063	0.008	0.501	0.026	0.054	/	0.019	0.007	0.158	0.008	/	/	0.257	/
PFPeS	0.0004	/	0.001	/	0.001	/	0.001	/	0.0007	/	0.0008	/	0.001	/	0.024	/	0.006	/	0.001	/	/	/	/	/	0.015	/
PFHxS	0.008	/	0.012	/	0.026	/	0.012	/	0.010	/	0.029	/	0.016	/	0.486	/	0.606	/	0.051	0.005	0.024	/	/	/	0.358	/
PFHpS	0.004	/	0.018	0.0009	0.198	/	0.022	0.001	0.014	/	0.005	/	0.005	/	0.162	/	0.124	/	0.036	/	0.011	/	0.046	0.060	0.152	/
PFOS	1.68	0.021	1.04	0.028	1.33	0.017	1.01	0.021	5.36	0.006	0.322	0.002	0.627	0.004	12.2	0.076	15.4	0.056	8.39	0.018	3.85	0.058	29.0	2.37	5.66	0.045
PFDS	0.028	/	0.028	/	0.020	/	0.025	/	0.048	/	0.017	/	0.080	/	2.24	/	1.03	/	0.405	/	0.139	/	0.147	0.098	0.046	/
FBSA	0.004	/	0.018	0.004	0.023	/	0.013	0.005	0.002	/	0.009	/	0.012	/	0.154	0.007	0.087	0.006	0.085	0.008	0.374	0.005	/	/	0.008	/
4:2 FTS	/	/	/	/	/	/	/	/	/	/	0.003	/	/	/	/	/	0.001	/	/	/	/	/	/	/	/	/
6:2 FTS	0.011	0.007	0.005	/	0.003	0.003	0.005	/	0.011	0.004	0.006	0.008	0.098	/	0.014	0.008	0.011	0.026	0.0007	0.001	/	/	0.670	0.567	0.015	0.014
8:2 FTS	/	/	/	/	/	/	/	/	/	/	/	/	0.0009	/	0.001	/	0.006	0.004	0.0007	/	/	/	/	/	/	/
NaDONA	/	/	/	/	/	/	/	/	/	/	0.0001	0.0002	/	/	/	/	/	/	/	/	/	/	/	/	0.002	0.002

Table S20: Relative standard deviation (%) based on the mean concentrations found in each matrix

Compound	Soil L1		Soil L3		Soil L5		Soil L7		Soil L10		Nettles		Isopods		Earthworms		Snails		Slugs		Spiders		Plasma		Feathers	
	BD3M	VB	BD3M	VB	BD3M	VB	BD3M	VB	BD3M	VB	BD3M	VB	BD3M	VB	BD3M	VB	BD3M	VB	BD3M	VB	BD3M	VB	BD3M	VB	BD3M	VB
PFBA	129	160	143	187	446	191	108	163	130	282	106	263	132	284	86.9	118	85.5	135	97.5	/	197	/	/	/	102.5	/
PFPeA	161	126	203	/	436	/	170	77.3	229	/	148	/	152	/	213	253	120	/	111	/	224	/	/	/	137.2	/
PFHxA	158	88.7	172	/	414	119	166	/	233	136	155	/	132	54.0	195	129	178	113	108	220	258	80.7	237	220	133.6	120.3
PFHpA	179	/	156	/	354	/	155	/	204	/	150	/	242	/	278	215	83.4	/	203	/	214	/	/	/	143.5	/
PFOA	174	86.2	154	74.4	344	65.2	143	74.5	217	96.1	150	62.2	138	70.5	183	345	164	70.9	116	64.0	103	62.7	145	123	134.3	67.2
PFNA	108	70.4	129	81.0	115	104	108	103	378	106	153	94.4 /	182	/	168	210	150	107	94.2	/	200	/	154	/	116.2	/
PFDA	447	43.1	412	/	328	57.8	386	/	387	23.6	50.4	56.9	106	34.6	138	62.5	121	69.6	81.5	61.1	94.3	34.7	64.7	67.2	76.9	57.6
PFUnDA	150	85.4	166	/	198	115	156	/	162	113	256	141	77.9	41.3	242	119	149	84.7	230	182	94.4	56.4	162	/	71.4	65.4
PFDoDA	167	63.7	155	180	155	73.7	190	296	149	129	283	150	186	26.9	182	61.7	115	114	156	153	194	46.5	135	282	190.6	74.1
PFTrDA	126	108	167	/	147	157	189	/	156	130	269	132	141	84.9	156	58.2	119	143	111	153	204	143	138	385	135.1	/
PFTeDA	139	/	176	/	170	/	175	/	163	/	238	/	139	187	168	104	126	151	106	158	179	/	263	/	171.6	/
PFBS	193	79.0	147	230	221	/	106	120	196	391	168	/	143	373	172	94.5	138	/	157	305	164	230	/	/	108.1	/
PFPeS	209	/	145	/	519	/	186	/	403	/	151	/	225	/	202	/	166	/	180	/	/	/	/	/	220.3	/
PFHxS	342	/	141	/	361	/	152	/	491	/	197	/	487	/	245	/	198	/	128	317	187	/	/	/	195.8	/
PFHpS	186	/	160	221	577	/	143	141	365	/	186	/	314	/	135	/	188	/	139	/	224	/	333	386	140.2	/
PFOS	139	78.2	132	57.9	135	104	125	56.3	401	62.3	126	116	164	172	139	135	180	123	104	63.0	189	230	114	377	119.6	60.9
PFDS	192	/	164	/	157	/	209	/	390	/	341	/	286	/	223	/	164	/	145	/	230	/	352	387	142.8	/
FBSA	151	/	108	83.5	533	/	99.9	105	272	/	149	/	151	/	94.2	107	72.5	60.6	79.8	94.8	189	106	/	/	156.2	/
4:2 FTS	/	/	/	/	/	/	/	/	/	/	210	/	/	/	/	/	187	/	/	/	/	/	/	/	/	/
6:2 FTS	170	149	298	/	208	66.9	246	/	261	145	303	204	449	/	257	386	147	157	157	313	/	/	479	387	247.9	177.5
8:2 FTS	/	/	/	/	/	/	/	/	/	/	/	/	472	/	275	/	213	235	164	/	/	/	/	/	/	/
NaDONA	/	/	/	/	/	/	/	/	/	/	89.0	80.3	/	/	/	/	/	/	/	/	/	/	/	/	138.3	117.3

Table S21 Results of pearson (p) or spearman (s) correlations made between PFAS in soil and PFAS in nettles found at Blokkersdijk/3M (BD3M), Vlietbos (VB) and both sites combined (BD3M-VB), with significant correlations in bold

Soil – Nettles	BD3M	VB	BD3M-VB
PFBA	p: r(21) = 0.06, p = 0.793	/	p: r(22) = 0.20, p = 0.345
PFPeA	p: r(12) = 0.42, p = 0.131	/	/
PFOA	**s: r(28) = 0.77, p = $2.01x10^{-6}$**	s: r(10) = 0.10, p = 0.761	**s: r(40) = 0.79, p = $4.43x10^{-10}$**
PFDA	**s: r(26) = 0.47, p = 0.012**	p: r(9) = 0.17, p = 0.610	**s: r(37) = 0.61, p = $4.10x10^{-5}$**
PFDoDA	**p: r(16) = 0.78, p = 0.0001**	/	**p: r(19) = 0.83, p = $3.70x10^{-6}$**
PFOS	**s: r(28) = 0.66, p = 0.0001**	**s: r(10) = -0.73, p = 0.010**	**s: r(40) = 0.82, p < $2.20x10^{-6}$**

Table S22: Results of pearson (p) or spearman (s) correlations made between PFAS in soil and PFAS in terrestrial invertebrates found at Blokkersdijk/3M (BD3M), Vlietbos (VB) and both sites combined (BD3M-VB)

	BD3M	VB	BD3M-VB
	Soil – Isopods		
PFBA	p: r(17) = -0.34, p = 0.148	/	p: r(18) = -0.15, p = 0.515
PFPeA	**s: r(15) = 0.68, p = 0.003**	/	/
PFHxA	**s: r(22) = 0.71, p = 0.0001**	/	**s: r(30) = 0.68, p = $1.87x10^{-5}$**
PFOA	**s: r(29) = 0.54, p = 0.002**	s: r(11) = 0.54, p = 0.061	**s: r(42) = 0.66, p = $2.10x10^{-6}$**
PFDA	s: r(29) = 0.22, p = 0.234	s: r(11) = 0.05, p = 0.872	s: r(42) = 0.24, p = 0.114
PFUnDA	s: r(29) = 0.08, p = 0.651	/	**s: r(37) = 0.43, p = 0.006**
PFDoDA	**s: r(27) = 0.61, p = 0.0004**	/	**s: r(37) = 0.72, p = $3.08x10^{-7}$**
PFTrDA	**s: r(25) = 0.68, p = 0.0001**	/	**s: r(28) = 0.73, p = $8.03x10^{-6}$**
PFTeDA	s: r(9) = 0.39, p = 0.237	/	/
PFOS	**s: r(30) = 0.79, p = $9.97x10^{-8}$**	p: r(10) = 0.07, p = 0.838	**s: r(42) = 0.90, p < $2.2x10^{-16}$**

Table S22 continued: Results of pearson (p) or spearman (s) correlations made between PFAS in soil and PFAS in terrestrial invertebrates found at Blokkersdijk/3M (BD3M), Vlietbos (VB) and both sites combined (BD3M-VB)

Soil – Earthworms			
PFBA	p: r(18) = 0.03, p = 0.891	/	p: r(22) = 0.326, p =0.120
PFPeA	**s: r(13) = 0.77, p = 0.001**	/	/
PFHxA	**s: r(17) = 0.70, p = 0.001**	/	**s: r(20) = 0.71, p = 0.0003**
PFOA	**s: r(21) = 0.64, p = 0.001**	s: r(12) = -0.02, p = 0.964	**s: r(35) = 0.83, p = $5.62x10^{-8}$**
PFNA	**s: r(15) = 0.73, p = 0.001**	/	**p: r(18) = 0.72, p = 0.0003**
PFDA	**s: r(19) = 0.51, p = 0.017**	p: r(13) = -0.16, p = 0.570	**s: r(34) = 0.60, p = $9.70x10^{-5}$**
PFUnDA	**s: r(18) = 0.48, p = 0.033**	p: r(3) = 0.33, p = 0.588	**s: r(23) = 0.59, p = 0.002**
PFDoDA	**s: r(20) = 0.73, p = 0.0002**	p: r(9) = 0.08, p = 0.814	**s: r(31) = 0.81, p = $5.21x10^{-7}$**
PFTrDA	**s: r(18) = 0.73, p = 0.0004**	/	**s: r(24) = 0.82, p = $2.15x10^{-6}$**
PFTeDA	**p: r(11) = 0.89, p = $4.68x10^{-5}$**	/	/
PFOS	**p: r(22) = 0.66, p = 0.0004**	s: r(12) = 0.12, p = 0.693	**s: r(36) = 0.88, p < $2.20x10^{-16}$**
Soil – Snails			
PFBA	p: r(10) = 0.56, p = 0.056	/	**s: r(12) = 0.75, p = 0.003**
PFHxA	p: r(5) = 0.23, p = 0.615	/	s: r(7) = 0.13, p = 0.744
PFOA	s: r(11) = 0.40, p = 0.180	p: r(5) = -0.08, p = 0.858	**s: r(18) = 0.72, p = 0.0004**
PFNA	**p: r(9) = 0.83, p = 0.002**	p: r(2) = -0.38, p = 0.625	**p: r(13) = 0.80, p = 0.0003**
PFDA	s: r(9) = 0.43, p = 0.189	**p: r(4) = 0.83, p = 0.040**	**p: r(15) = 0.64, p = 0.006**
PFUnDA	**p: r(8) = 0.86, p = 0.001**	/	**p: r(10) = 0.88, p = 0.0002**
PFDoDA	**s: r(11) = 0.75, p = 0.005**	p: r(3) = -0.05, p = 0.939	**s: r(16) = 0.80, p = $9.34x10^{-5}$**
PFTrDA	p: r(10) = 0.41, p = 0.191	/	**s: r(11) = 0.64, p = 0.021**
PFTeDA	p: r(5) = 0.14, p = 0.768	/	/
PFOS	**p: r(11) = 0.62, p = 0.023**	p: r(5) = 0.20, p = 0.665	**s: r(18) = 0.85, p < $2.20x10^{-16}$**
6:2 FTS	p: r(3) = 0.16, p = 0.799	p: r(1) = 0.45, p = 0.711	p: r(6) = 0.22, p = 0.595

Table S22 continued: Results of pearson (p) or spearman (s) correlations made between PFAS in soil and PFAS in terrestrial invertebrates found at Blokkersdijk/3M (BD3M), Vlietbos (VB) and both sites combined (BD3M-VB)

Soil – Slugs			
PFBA	p: r(12) = -0.11, p = 0.711	/	/
PFHxA	**p: r(6) = 0.72, p = 0.045**	/	/
PFOA	**s: r(13) = 0.80, p = 0.0005**	/	**s: r(22) = 0.87, p = 2.46×10^{-6}**
PFNA	s: r(11) = 0.35, p = 0.239	/	/
PFDA	**s: r(11) = 0.60, p = 0.031**	/	s: r(22) = 0.27, p = 0.198
PFUnDA	**s: r(8) = 0.72, p = 0.018**	/	/
PFDoDA	**s: r(12) = 0.89, p < 2.20×10^{-16}**	/	**s: r(15) = 0.85, p < 2.20×10^{-16}**
PFTrDA	p: r(10) = 0.51, p = 0.087	/	/
PFTeDA	p: r(4) = 0.66, p = 0.158	/	/
PFOS	**s: r(13) = 0.67, p = 0.008**	s: r(9) = 0.26, p = 0.435	**s: r(24) = 0.88, p = 1.75×10^{-6}**
Soil – Spiders			
PFHxA	/	p: r(3) = -0.53, p = 0.361	s: r(6) = -0.07, p = 0.882
PFOA	s: r(7) = 0.31, p = 0.462	p: r(3) = 0.28, p = 0.649	**s: r(11) = 0.78, p = 0.003**
PFDA	s: r(7) = 0.27, p = 0.493	p: r(3) = -0.09, p = 0.882	s: r(12) = 0.49, p = 0.078
PFUnDA	p: r(5) = -0.14, p = 0.773	p: r(2) = -0.47, p = 0.527	s: r(9) = 0.16, p = 0.634
PFDoDA	/	p: r(3) = -0.80, p = 0.102	s: r(8) = 0.53, p = 0.123
PFOS	**p: r(7) = 0.72, p = 0.030**	p: r(3) = -0.42, p = 0.485	**p: r(12) = 0.84, p = 0.0002**

Table S23: Results of pearson (p) or spearman (s) correlations made between PFAS in soil and PFAS in great tits sampled at Blokkersdijk/3M (BD3M), Vlietbos (VB) and both sites combined (BD3M-VB)

Topsoil – Great tits	BD3M	VB	BD3M-VB
Topsoil – Plasma			
PFOA	p: r(17) = 0.22, p = 0.362	**s: r(12) = 0.64, p = 0.014**	**s: r(31) = 0.54, p = 0.001**
PFDA	s: r(17) = 0.16, p = 0.522	s: r(13) = 0.17, p = 0.547	**s: r(32) = 0.48, p = 0.004**
PFDoDA	**s: r(17) = 0.51, p = 0.026**	/	/
PFTrDA	s: r(13) = 0.15, p = 0.584	/	/
PFOS	s: r(17) = 0.21, p = 0.397	/	/
Topsoil – Feathers			
PFBA	s: r(16) = -0.12, p = 0.636	/	
PFPeA	s: r(13) = -0.28, p = 0.314	/	
PFHxA	s: r(16) = 0.39, p = 0.106	/	
PFOA	p: r(16) = 0.30, p = 0.226	s: r(12) = 0.20, p = 0.497	**s: r(30) = 0.61, p = 0.0002**
PFDA	s: r(16) = 0.25, p = 0.316	/	s: r(31) = 0.09, p = 0.612
PFUnDA	s: r(15) = 0.40, p = 0.111	s: r(11) = -0.07, p = 0.809	s: r(28) = 0.13, p = 0.494
PFDoDA	**s: r(16) = 0.66, p = 0.003**	s: r(12) = -0.13, p = 0.657	**s: r(30) = 0.47, p = 0.006**
PFOS	**s: r(16) = 0.62, p = 0.006**	s: r(13) = 0.03, p = 0.919	**s: r(31) = - 0.49, p = 0.004**

Table S24: Results of pearson (p) or spearman (s) correlations made between PFAS in nettles and PFAS in terrestrial invertebrates found at Blokkersdijk/3M (BD3M), Vlietbos (VB) and both sites combined (BD3M-VB)

Nettles – Invertebrates	BD3M	VB	BD3M-VB
Nettles – Isopods			
PFBA	p: r(20) = -0.10, p = 0.666	/	/
PFPeA	**s: r(17) = 0.68, p = 0.002**	/	/
PFOA	**s: r(33) = 0.59, p = 0.0002**	p: r(9) = -0.14, p = 0.691	**s: r(44) = 0.60, p = $1.05x10^{-5}$**
PFDA	**p: r(30) = 0.44, p = 0.011**	**p: r(10) = -0.61, p = 0.036**	s: r(42) = 0.14, p = 0.364
PFDoDA	**s: r(23) = 0.73, p = $5.21x10^{-5}$**	/	/
PFOS	**s: r(32) = 0.82, p = $2.31x10^{-9}$**	s: r(10) = -0.43, p = 0.161	**s: r(44) = 0.88, p = $1.28x10^{-15}$**
Nettles – Earthworms			
PFBA	p: r(20) = -0.22, p = 0.323	/	p: r(21) = 0.02, p = 0.938
PFPeA	p: r(10) = 0.53, p = 0.075	/	/
PFOA	**s: r(25) = 0.55, p = 0.004**	s: r(10) = 0.15, p = 0.648	**s: r(37) = 0.67, p = $3.86x10^{-6}$**
PFDA	**p: r(21) = 0.50, p = 0.015**	p: r(10) = -0.36, p = 0.253	**s: r(33) = 0.42, p = 0.012**
PFDoDA	**p: r(17) = 0.72, p = 0.0005**	/	/
PFOS	**s: r(25) = 0.55, p = 0.003**	s: r(11) = 0.05, p = 0.878	**s: r(38) = 0.80, p = $3.13x10^{-8}$**
Nettles – Snails			
PFBA	p: r(10) = -0.01, p = 0.972	/	p: r(11) = 0.12, p = 0.690
PFOA	s: r(12) = 0.37, p = 0.199	p: r(3) = -0.03, p = 0.960	**s: r(17) = 0.56, p = 0.013**
PFDA	p: r(9) = 0.57, p = 0.069	s: r(2) = 0.20, p = 0.917	**p: r(13) = 0.64, p = 0.010**
PFDoDA	s: r(7) = 0.10, p = 0.810	/	/
PFOS	p: r(12) = 0.47, p = 0.093	p: r(4) = 0.41, p = 0.416	**s: r(18) = 0.69, p = 0.001**

Table S24 continued: Results of pearson (p) or spearman (s) correlations made between PFAS in nettles and PFAS in terrestrial invertebrates found at Blokkersdijk/3M (BD3M), Vlietbos (VB) and both sites combined (BD3M-VB)

Nettles – Slugs			
PFBA	p: r(13) = -0.14, p = 0.606	/	/
PFOA	**s: r(13) = 0.82, p = 0.0003**	/	/
PFDA	**p: r(14) = 0.68, p = 0.004**	/	/
PFDoDA	**p: r(7) = 0.80, p = 0.010**	/	/
PFOS	**s: r(14) = 0.89, p < 2.20×10^{-6}**	s: r(9) = -0.23, p = 0.503	**s: r(25) = 0.90, p = 1.12×10^{-6}**
Nettles – Spiders			
PFOA	s: r(7) = 0.57, p = 0.121	p: r(2) = -0.65, p = 0.345	**s: r(11) = 0.73, p = 0.005**
PFDA	p: r(6) = -0.15, p = 0.731	p: r(4) = -0.47, p = 0.345	p: r(12) = -0.05, p = 0.857
PFOS	**p: r(7) = 0.83, p = 0.006**	**p: r(2) = 0.97, p = 0.034**	**p: r(11) = 0.81, p = 0.0009**

Table S25: Results of pearson (p) or spearman (s) correlations made between PFAS in nettles and PFAS in great tits sampled at Blokkersdijk/3M (BD3M), Vlietbos (VB) and both sites combined (BD3M-VB)

Nettles – Great tits	BD3M	VB	BD3M-VB
Nettles – Plasma			
PFOA	s: r(21) = 0.30, p = 0.162	s: r(13) = 0.17, p = 0.646	**s: r(36) = 0.48, p = 0.002**
PFDA	s: r(19) = 0.27, p = 0.231	s: r(12) = -0.22, p = 0.475	**s: r(34) = 0.42, p = 0.013**
PFDoDA	s: r(20) = -0.01, p = 0.962	/	/
PFOS	s: r(21) = 0.21, p = 0.329	/	/
Nettles – Feathers			
PFBA	s: r(21) = -0.31, p = 0.148	/	/
PFPeA	s: r(21) = -0.24, p = 0.271	/	/
PFOA	s: r(21) = 0.14, p = 0.526	s: r(13) = 0.13, p = 0.646	**s: r(36) = 0.49, p = 0.002**
PFDA	**s: r(19) = 0.47, p = 0.030**	s: r(13) = 0.30, p = 0.284	s: r(34) = 0.27, p = 0.106
PFDoDA	s: r(17) = 0.31, p = 0.191	/	/
PFOS	s: r(21) = 0.38, p = 0.072	/	/

Table S26: Results of pearson (p) or spearman (s) correlations made between PFAS in terrestrial invertebrates and PFAS in great tit plasma sampled at Blokkersdijk/3M (BD3M), Vlietbos (VB) and both sites combined (BD3M-VB)

Invertebrates – Plasma	BD3M	VB	BD3M-VB
Isopods – Plasma			
PFOA	**s: r(20) = 0.56, p = 0.007**	s: r(12) = 0.23, p = 0.436	**s: r(34) = 0.52, p = 0.001**
PFDA	s: r(20) = 0.10, p = 0.649	s: r(12) = 0.34, p = 0.228	s: r(34) = 0.21, p = 0.211
PFDoDA	s: r(20) = 0.24, p = 0.278	/	/
PFTrDA	s: r(10) = 0.05, p = 0.873	/	/
PFOS	s: r(20) = 0.16, p = 0.472	/	/
Earthworms – Plasma			
PFOA	**s: r(17) = 0.47, p = 0.044**	s: r(13) = -0.02, p = 0.934	**s: r(32) = 0.47, p = 0.005**
PFDA	**s: r(16) = 0.61, p = 0.008**	s: r(13) = -0.05, p = 0.873	**s: r(31) = 0.51, p = 0.002**
PFDoDA	s: r(17) = 0.33, p = 0.167	/	/
PFTrDA	**s: r(17) = 0.55, p = 0.015**	/	/
PFOS	s: r(17) = 0.17, p = 0.492	/	/
Snails – Plasma			
PFOA	s: r(12) = 0.44, p = 0.118	p: r(4) = -0.12, p = 0.825	s: r(18) = 0.43, p = 0.06
PFDA	s: r(12) = 0.44, p = 0.117	s: r(5) = -0.43, p = 0.335	**s: r(19) = 0.47, p = 0.031**
PFDoDA	s: r(10) = 0.42, p = 0.173	/	/
PFTrDA	s: r(10) = 0.54, p = 0.070	/	/
PFOS	s: r(12) = 0.17, p = 0.572	/	/
Slugs – Plasma			
PFOA	**s: r(15) = 0.60, p = 0.011**	/	/
PFDA	s: r(15) = 0.18, p = 0.484	/	/
PFDoDA	s: r(13) = 0.27, p = 0.333	/	/
PFTrDA	s: r(9) = 0.16, p = 0.638	/	/
PFOS	**p: r(15) = 0.51, p = 0.038**	/	/

Table S26 continued: Results of pearson (p) or spearman (s) correlations made between PFAS in terrestrial invertebrates and PFAS in great tit plasma sampled at Blokkersdijk/3M (BD3M), Vlietbos (VB) and both sites combined (BD3M-VB)

Spiders – Plasma			
PFOA	s: r(9) = -0.22, p = 0.516	p: r(5) = 0.15, p = 0.811	s: r(16) = 0.32, p = 0.191
PFDA	s: r(9) = 0.20, p = 0.552	s: r(5) = -0.30, p = 0.519	s: r(16) = 0.25, p = 0.316
PFOS	s: r(9) = 0.17, p = 0.609	/	/

Table S27: Results of pearson (p) or spearman (s) correlations made between PFAS in terrestrial invertebrates and PFAS in great tit feathers sampled at Blokkersdijk/3M (BD3M), Vlietbos (VB) and both sites combined (BD3M-VB)

Invertebrates – Feathers	BD3M	VB	BD3M-VB
Isopods – Feathers			
PFBA	s: r(18) = -0.26, p = 0.263	/	/
PFPeA	s: r(15) = 0.05, p = 0.848	/	/
PFHxA	s: r(20) = -0.02, p = 0.922	/	/
PFOA	s: r(20) = 0.17, p = 0.457	s: r(12) = 0.25, p = 0.382	**s: r(34) = 0.37, p = 0.026**
PFDA	s: r(20) = -0.12, p = 0.588	s: r(12) = 0.45, p = 0.107	s: r(34) = -0.01, p = 0.953
PFDoDA	**s: r(20) = 0.54, p = 0.009**	p: r(12) = 0.19, p = 0.656	**s: r(34) = 0.43, p = 0.010**
PFUnDA	s: r(20) = 0.31, p = 0.161	p: r(12) = 0.28, p = 0.331	s: r(34) = 0.22, p = 0.196
PFBS	s: r(18) = -0.44, p = 0.05	/	/
PFOS	s: r(20) = 0.46, p = 0.033	p: r(12) = 0.40, p = 0.160	**s: r(34) = 0.80, p = 4.27x10^{-9}**
Earthworms – Feathers			
PFBA	s: r(16) = -0.28, p = 0.266	/	/
PFPeA	s: r(14) = -0.03, p = 0.914	/	/
PFHxA	s: r(16) = 0.24, p = 0.342	/	/
PFOA	s: r(17) = 0.20, p = 0.415	s: r(13) = 0.19, p = 0.504	**s: r(32) = 0.56, p = 0.0005**
PFDA	s: r(17) = 0.39, p = 0.101	p: r(13) = 0.20, p = 0.484	s: r(32) = 0.30, p = 0.09
PFDoDA	**s: r(17) = 0.57, p = 0.011**	s: r(13) = 0.20, p = 0.468	s: r(32) = 0.48, p = 0.004
PFUnDA	s: r(16) = 0.19, p = 0.470	s: r(10) = -0.15, p = 0.631	s: r(27) = 0.01, p = 0.966
PFBS	s: r(15) = 0.06, p = 0.811	/	/
PFOS	s: r(17) = 0.32, p = 0.186	s: r(13) = -0.09, p = 0.755	s: r(32) = 0.75, p = 4.32x10^{-7}

Table S27 continued: Results of pearson (p) or spearman (s) correlations made between PFAS in terrestrial invertebrates and PFAS in great tit feathers sampled at Blokkersdijk/3M (BD3M), Vlietbos (VB) and both sites combined (BD3M-VB)

Snails – Feathers			
PFBA	s: r(12) = -0.44, p = 0.119	/	/
PFOA	**p: r(12) = 0.56, p = 0.037**	p: r(5) = -0.75, p = 0.05	**s: r(18) = 0.56, p = 0.008**
PFDA	s: r(12) = 0.34, p = 0.235	**p: r(5) = -0.94, p = 0.001**	s: r(19) = 0.005, p = 0.984
PFDoDA	**s: r(10) = 0.79, p = 0.002**	s: r(5) = -0.62, p = 0.140	s: r(17) = 0.44, p = 0.061
PFUnDA	s: r(12) = 0.32, p = 0.269	/	/
PFBS	s: r(10) = -0.32, p = 0.312	/	/
PFOS	s: r(12) = 0.17, p = 0.023	p: r(5) = -0.68, p = 0.093	**p: r(19) = 0.69, p = 0.0006**
Slugs – Feathers			
PFBA	s: r(15) = 0.04, p = 0.881	/	/
PFHxA	s: r(14) = -0.06, p = 0.832	/	/
PFOA	p: r(15) = 0.18, p = 0.491	/	/
PFDA	s: r(15) = 0.38, p = 0.135	/	/
PFDoDA	s: r(13) = 0.39, p = 0.148	/	/
PFUnDA	s: r(12) = 0.15, p = 0.618	/	/
PFOS	s: r(15) = 0.36, p = 0.160	s: r(9) = 0.08, p = 0.806	**s: r(26) = 0.77, p = 1.96x10^{-6}**
Spiders – Feathers			
PFOA	p: r(9) = -0.28, p = 0.410	p: r(5) = 0.57, p = 0.183	s: r(16) = 0.27, p = 0.276
PFDA	s: r(9) = 0.11, p = 0.758	p: r(5) = 0.11, p = 0.815	s: r(16) = -0.13, p = 0.620
PFDoDA	/	p: r(5) = -0.07, p = 0.875	/
PFUnDA	s: r(9) = 0.06, p = 0.852	p: r(5) = -0.24, p = 0.606	s: r(16) = -0.10, p = 0.698
PFBS	s: r(7) = -0.56, p = 0.1145	/	/
PFOS	s: r(9) = -0.25, p = 0.455	s: r(5) = -0.52, p = 0.233	**s: r(16) = 0.60, p = 0.008**

Table S28: Results of pearson (p) or spearman (s) correlations made between PFAS in plasma and PFAS in feathers of great tits sampled at Blokkersdijk/3M (BD3M), Vlietbos (VB) and both sites combined (BD3M-VB)

Plasma – feathers	BD3M	VB	BD3M-VB
PFOA	s: r(17) = 0.37, p = 0.133	s: r(7) = -0.38, p = 0.360	**p: r(25) = 0.40, p = 0.045**
PFDA	s: r(13) = -0.40, p = 0.176	s: r(9) = -0.07, p = 0.865	s: r(22) = -0.159, p = 0.467
PFDoDA	s: r(9) = 0.45, p = 0.191	/	s: r(12) = 0.379, p = 0.202
PFOS	**p: r(17) = 0.63, p = 0.005**	/	**p: r(22) = 0.60, p = 0.002**
